# The CENP-T/-W complex is a binding partner of the histone chaperone FACT

**DOI:** 10.1101/gad.275073.115

**Published:** 2016-06-01

**Authors:** Lisa Prendergast, Sebastian Müller, Yiwei Liu, Hongda Huang, Florent Dingli, Damarys Loew, Isabelle Vassias, Dinshaw J. Patel, Kevin F. Sullivan, Geneviève Almouzni

**Affiliations:** 1 UMR3664, Centre National de la Recherche Scientifique, Institut Curie, PSL (Paris Sciences et Lettres) Research University, F-75005 Paris, France;; 2 UMR3664, Centre National de la Recherche Scientifique, University Pierre and Marie Curie Paris 06, Sorbonne Universités, F-75005 Paris, France;; 3 Structural Biology Program, Memorial Sloan-Kettering Cancer Center, New York, New York 10065, USA;; 4 Laboratoire de Spectrométrie de Masse Protéomique, Institut Curie, PSL (Paris Sciences et Lettres) Research University Centre de Recherche, Paris 75005, France;; 5 Centre for Chromosome Biology, School of Natural Sciences, National University of Ireland, Galway, Ireland

**Keywords:** centromere, CENP, histone chaperone, mitosis

## Abstract

The CENP-T/-W histone fold complex, as an integral part of the inner kinetochore, is essential for building a proper kinetochore at the centromere in order to direct chromosome segregation during mitosis. Notably, CENP-T/-W is not inherited at centromeres, and new deposition is absolutely required at each cell cycle for kinetochore function. However, the mechanisms underlying this new deposition of CENP-T/-W at centromeres are unclear. Here, we found that CENP-T deposition at centromeres is uncoupled from DNA synthesis. We identified Spt16 and SSRP1, subunits of the H2A–H2B histone chaperone facilitates chromatin transcription (FACT), as CENP-W binding partners through a proteomic screen. We found that the C-terminal region of Spt16 binds specifically to the histone fold region of CENP-T/-W. Furthermore, depletion of Spt16 impairs CENP-T and CENP-W deposition at endogenous centromeres, and site-directed targeting of Spt16 alone is sufficient to ensure local de novo CENP-T accumulation. We propose a model in which the FACT chaperone stabilizes the soluble CENP-T/-W complex in the cell and promotes dynamics of exchange, enabling CENP-T/-W deposition at centromeres.

## Abstract (precis)

Prendergast et al. identified Spt16 and SSRP1, subunits of the H2A–H2B histone chaperone FACT, as CENP-W-binding partners through a proteomic screen. They developed a model in which the FACT chaperone stabilizes the soluble CENP-T/-W complex in the cell and promotes dynamics of exchange, enabling CENP-T/-W deposition at centromeres.

The centromere is a specialized chromosomal locus that defines the site of kinetochore assembly. Kinetochore assembly occurs in each cell cycle to direct chromosome segregation during cell division. The functional identity of the centromere is thought to be conveyed by the histone variant CenH3^CENP-A^ (for review, see Black and Cleveland 2011; Müller and Almouzni 2014). This foundation enables nucleation of a core group of 17 additional proteins, the constitutive centromere-associated network (CCAN) necessary for kinetochore function (Foltz et al. 2006; Izuta et al. 2006; Okada et al. 2006). Crucially, this chromatin-associated complex recruits the KNL1/Mis12 complex/Ndc80 complex (KMN) network, which acts as the primary microtubule-binding interface at kinetochores (Cheeseman et al. 2006). Thus, the CCAN functions as a regulator of mitosis through the coordination of the spindle assembly checkpoint and error correction mechanisms (Santaguida and Musacchio 2009; Gascoigne and Cheeseman 2011). The CENP-T/-W complex is a core component of the CCAN whose presence at centromeres is critical for kinetochore assembly. CENP-T plays key roles both in recruiting downstream CCAN components and directly binding to Ndc80, the conserved microtubule-binding complex of kinetochores (Gascoigne et al. 2011; Hori et al. 2012; Malvezzi et al. 2013). Notably, in contrast to CenH3^CENP-A^, CENP-T/-W is not retained at centromeres through cell division (Jansen et al. 2007; Prendergast et al. 2011). Therefore, a faithful de novo deposition of CENP-T/-W during each cell cycle is essential for centromere maturation and kinetochore formation in preparation for mitosis (Prendergast et al. 2011). While CENP-T and CENP-W de novo deposition occurs in S phase (Prendergast et al. 2011), the mechanisms controlling this new deposition are currently unknown. CENP-T/-W accumulation at centromeres is downstream from other CCAN components, including CENP-C (Carroll et al. 2010; Basilico et al. 2014; Tachiwana et al. 2015) and the CENP-H/-I/-K/-M complex (Basilico et al. 2014; Klare et al. 2015). CENP-T/-W does not directly interact with CenH3^CENP-A^ (Hori et al. 2008). Moreover, reduced CenH3^CENP-A^ levels do not impact CENP-T recruitment to centromeres (Fachinetti et al. 2013). Previous studies showed that CENP-T/-W forms a heterotetrameric particle in vitro with CENP-S and CENP-X, which in turn can bind centromeric DNA (Nishino et al. 2012; Takeuchi et al. 2013). However, the existence of this particle in vivo remains elusive. Furthermore, CENP-T/-W does not depend on CENP-S and CENP-X for centromere localization (Takeuchi et al. 2013). Post-translational modifications of CENP-T influence both its localization and interactions with kinetochore components. Indeed, particular phosphorylation by CDKs in humans or by the Mps1 kinase in *Saccharomyces cerevisiae* facilitates centromere targeting of CENP-T (Gascoigne and Cheeseman 2013; Thapa et al. 2015), while phosphorylation of CENP-T also regulates its interaction with the Ndc80 complex (Nishino et al. 2013). Thus, several functional interactions of the CENP-T/-W complex at centromeres have been characterized, yet the molecular mechanism enabling CENP-T/-W accumulation at centromeres remains unclear. CENP-T and CENP-W both contain C-terminal histone fold domains (HFDs), which mediate the formation of the CENP-T/-W heterodimer (Hori et al. 2008). The lack of this C-terminal HFD region in CENP-T impairs its localization to centromeres (Nishino et al. 2012; Thapa et al. 2015). HFDs have the ability to form stable protein–protein interactions and are often required for the binding of histones to their dedicated chaperones. Histone chaperones escort histones and prevent spurious nonspecific histone interactions; they can facilitate histone transfer in particular during transport from the cytosol into the nucleus and histone deposition to chromatin without themselves being part of the final product (Probst et al. 2009; Burgess and Zhang 2013; Gurard-Levin et al. 2013; Müller and Almouzni 2014). Here, we found that CENP-T deposition at centromeres does not depend on DNA synthesis. To gain insights into the CENP-T/-W deposition mechanism, we searched for putative CENP-T/-W-binding partners in a proteomic screen. Our proteomic analysis reveals that both subunits of the facilitates chromatin transcription (FACT) complex (namely, Spt16 and SSRP1) associate with CENP-W. FACT is known to be a H2A–H2B histone chaperone, which facilitates histone dynamics at chromatin in concert with polymerases involved in replication, transcription, and repair (Orphanides et al. 1999; Hondele and Ladurner 2011; Winkler and Luger 2011, Hsieh et al. 2013). FACT function is essential in vertebrates, as depletion is embryonic-lethal (Cao et al. 2003). In *S. cerevisiae*, depletion of the Spt16 FACT subunit inhibits cell growth (Deyter and Biggins 2014), and FACT plays a role in restricting the incorporation of CenH3^Cnp1^ at locations outside centromeres (Choi et al. 2012). Interestingly, impairing FACT function also impairs centromeric heterochromatin integrity (Lejeune et al. 2007) and centromeric CenH3^CENP-A^ accumulation in several model systems (Okada et al. 2009; Chen et al. 2015), thus impacting centromere function. However, the exact reasons for these defects remain unclear. Here, we demonstrate that FACT is in a complex with CENP-T/-W in vivo and can directly interact with CENP-T/-W in vitro. Biochemical analysis coupled with cellular fractionation further identifies FACT in a complex with CENP-T/-W in the absence of CENP-C and CENP-S, indicating that the FACT–CENP-T/-W complex forms independently of CENP-C and CENP-S. We reveal that a 39-amino-acid domain at the C terminus of Spt16, which recruits H2A–H2B dimers to FACT, is sufficient to directly bind the HFDs of CENP-T/-W. We show that while H2A–H2A or CENP-T/-W can directly bind Spt16, the histone fold complex CENP-S/-X does not. Furthermore, depletion of Spt16 diminishes the accumulation of CENP-T at endogenous centromeres and reduces CENP-T recruitment by a LacI-CENP-C fusion protein at a LacO array. Importantly, anchoring LacI–Spt16 is sufficient to directly stimulate de novo accumulation of CENP-T in vivo at the corresponding LacO site. Taken together, we propose that FACT acts as a histone chaperone that switches its associated partners between H2A–H2B and CENP-T/-W, regulating CENP-T/-W accumulation at centromeres.

## Results

### Centromeric assembly of the CENP-T/-W complex does not depend on DNA synthesis

**Figure 1.**
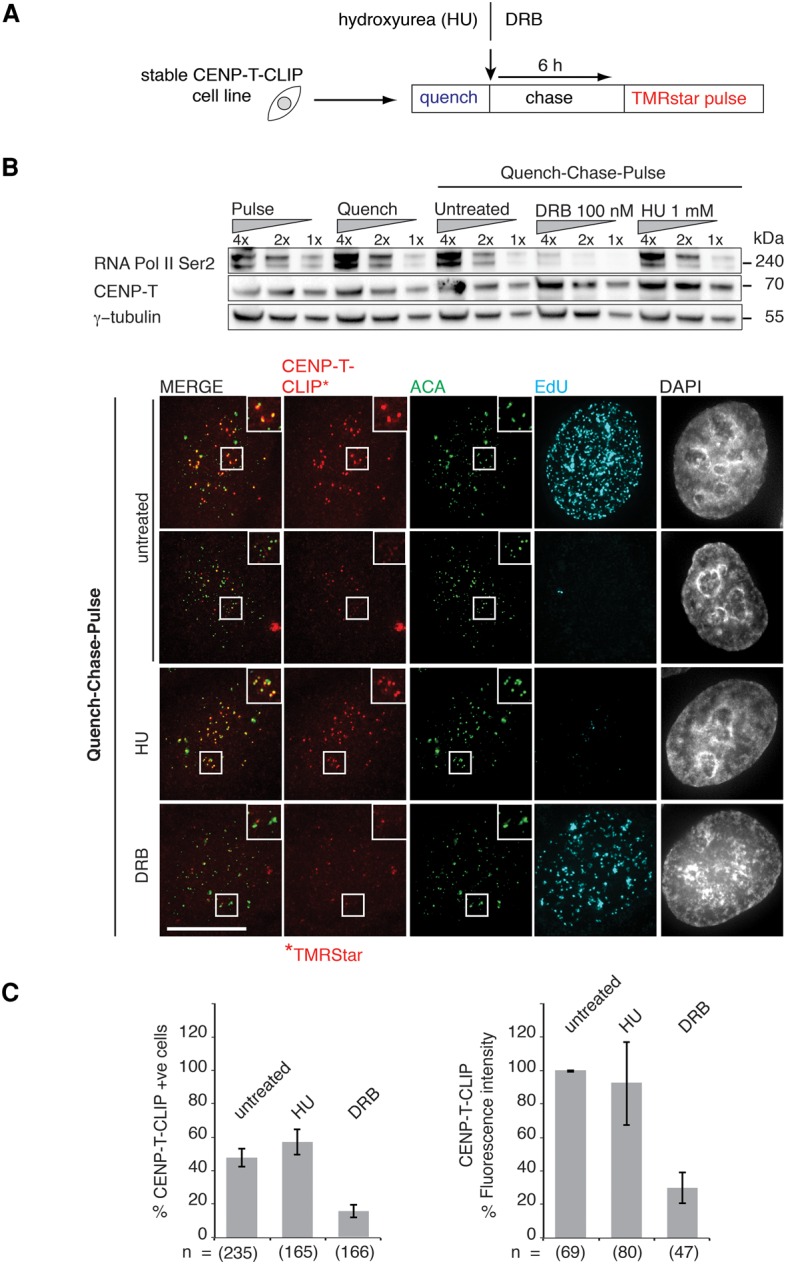
Assembly of newly synthesized CENP-T is uncoupled from DNA synthesis. (*A*) The experimental scheme outlines the quench–chase–pulse approach. A conditional in vivo labeling strategy was used, as previously described (Prendergast et al. 2011). (*B*) Cells expressing CLIP-tagged CENP-T in an asynchronous culture were labeled with CLIP cell block (New England Biolabs) to “quench” reactive CLIP proteins. A “chase” time was used to allow synthesis of “new” protein. The newly synthesized protein labeled with a “pulse” of fluorescent CLIP substrate. Final drug concentrations of 100 nM β-D-ribofuranosylbenzimidazole (DRB) and 1 mM hydroxyurea (HU) were added during the quench and maintained throughout the experiment. Western blot shows reduced levels of RNA polymerase II (Pol II) Ser2 in DRB-treated samples. BTC (immunofluorescence) with anti-centromere antibody (ACA) (green) was used to select centromeres. EdU (cyan) shows cells undergoing active DNA synthesis, while TMR-Star (red) was used to label new CENP-T-CLIP proteins. Bar, 10 µM. “Untreated” EdU-positive cells display assembly of newly synthesized CENP-T-CLIP at centromeres. EdU-negative cells do not show assembly. (*C*) The numbers of cells with detectable assembly (five CENP-T-CLIP-positive centromeres or more visible) were counted, and fluorescence intensities of centromeres were also measured. Approximately 43% of cells in a total untreated population exhibit newly synthesized CENP-T-CLIP signal at centromeres following a quench–chase–pulse assembly assay. In HU-treated samples, 56% of the population have newly synthesized CENP-T-CLIP signal at centromeres. In DRB-treated cells, 14% of the population have newly synthesized CENP-T-CLIP signal at centromeres. The fluorescence intensities of centromeres in EdU-positive cells are also reduced in the DRB-treated samples. Experiments were repeated three times, with a minimum of 30 S-phase cells counted for each experiment. *n* = number of cells. Error bars represent standard error of the mean (SEM).

The de novo loading of CENP-T and CENP-W occurs during S phase (Prendergast et al. 2011). Thus, it was important to assess whether this loading depends on DNA synthesis or other metabolic processes such as transcription. To address this issue, we used a “quench–chase–pulse” assay in cell lines stably expressing CENP-T-CLIP or CENP-W-CLIP (Prendergast et al. 2011) to test for effects on CENP-T or CENP-W accumulation at centromeres. The CLIP tag allows specific labeling of the tagged protein with a substrate; in this case, a fluorescent label (TMR-Star) or the blocking substrate bromothenylcytosine (BTC; BC-quench). Briefly, the “quench” step blocks all CENP-T present in the cell at the start of the experiment, the “chase” time allows for the synthesis of new protein, and the “pulse” labels only the newly synthesized CLIP-tagged protein (Fig. 1A). EdU was used to label cells undergoing DNA synthesis. Newly synthesized CENP-T-CLIP and CENP-W-CLIP accumulation at centromeres occurs in S phase; thus, most EdU-negative cells did not show CENP-T-CLIP or CENP-W-CLIP accumulation at centromeres, in agreement with previous reports (Fig. 1B; Supplemental Figs. S1b, S2b; Prendergast et al. 2011). In contrast, as expected, EdU-positive cells had significant CENP-T-CLIP accumulation at centromeres (Fig. 1B).

We then treated cells with either the RNA polymerase II (Pol II) Ser 2 phosphorylation inhibitor 5,6-dichloro-1-β-D-ribofuranosylbenzimidazole (DRB) or hydroxyurea (HU) to block DNA synthesis (Fig. 1B). DRB blocks the elongating form of RNA Pol II (RNA Pol II Ser2 phosphoform). The reduction in the RNA Pol II Ser2 phosphoform was confirmed by Western blot (Fig. 1B; Supplemental Figs. S1d, S2d). The effects on the cell cycle were monitored by FACS analysis (Supplemental Figs. S1a, S2a). We next tested whether de novo CENP-T-CLIP or CENP-W-CLIP synthesis was inhibited by DRB or HU treatment. SDS-PAGE analyses of CLIP cell TMR-Star-labeled protein samples allowed visualization and quantification of the newly synthesized fluorescent TMR-labeled protein (Supplemental Figs. S1c, S2c). Cycloheximide (CHX) was used to inhibit new protein synthesis as a control. In DRB- or HU-treated samples, the total protein levels of newly synthesized CENP-T-CLIP or CENP-W-CLIP were not significantly reduced (Supplemental Figs. S1c, S2c). We next quantified the fluorescence intensity of de novo deposited CENP-T-CLIP and CENP-W-CLIP at centromeres costained with either anti-centromere antibody (ACA) or CENP-B. Under DRB treatment, both CENP-T-CLIP (Fig. 1C; Supplemental Fig. S2d) and CENP-W-CLIP (Supplemental Fig. S1d) accumulation at centromeres was significantly impaired. In addition, fewer cells showed accumulation of CENP-T-CLIP at centromeres (Fig. 1C). Given that the total amount of newly synthesized CENP-T or CENP-W protein is not significantly reduced, this raises the possibility that transcription may play a role either directly or indirectly in de novo CENP-T and CENP-W centromere deposition. Strikingly, in HU-treated samples, in the absence of detectable DNA synthesis, CENP-T-CLIP deposition at centromeres is similar to untreated cells (Fig. 1B,C; Supplemental Fig. S2d). We obtained the same result with the cell line stably expressing CENP-W-CLIP (Supplemental Fig. S1d). Thus, our results suggest that ongoing DNA synthesis is not required for deposition of newly synthesized CENP-T or CENP-W at centromeres.

### FACT subunits interact with the CENP-T/-W complex

**Figure 2.**
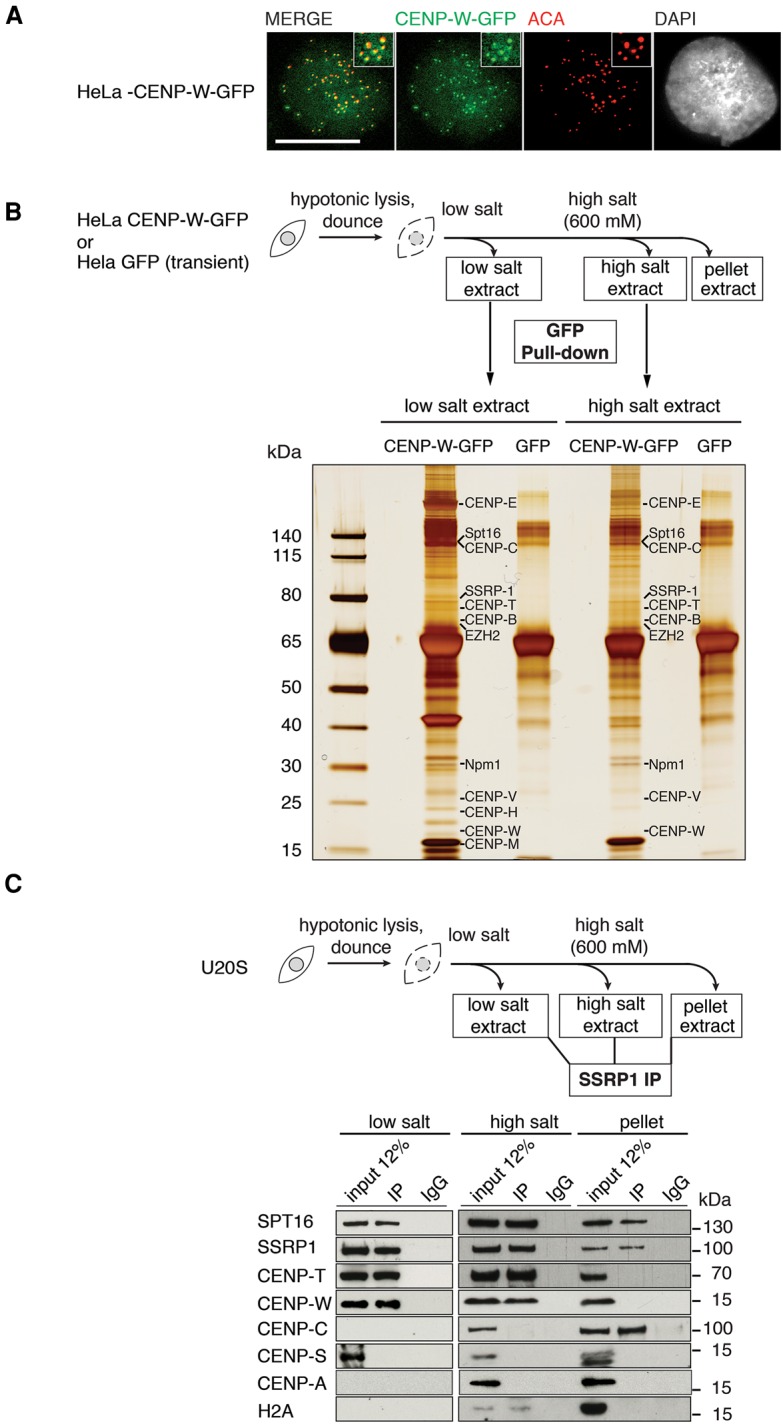
Isolation of CENP-W-GFP interactors. (*A*) A HeLa cell line stably expressing CENP-W-GFP was used to isolate complexes associated with CENP-W. The immunofluorescence panel shows colocalization of CENP-W-GFP with centromeres stained with ACA. (*B*) CENP-W-GFP complexes were isolated by pull-down of CENP-W-GFP from both low-salt and high-salt extracts using GFP-Trap. The silver-stained gel corresponds to complexes isolated in association with either GFP alone or CENP-W-GFP. Complexes purified with CENP-W-GFP were analyzed by MS. Peptides corresponding to several known proteins were identified as putative CENP-W interactors by MS. MS was performed on biological triplicates for samples and controls. (*C*) Western blot of SSRP1 co-IP from low-salt, high-salt, and pellet extracts prepared from U2OS cells. Spt16, CENP-T, and CENP-W coimmunoprecipitate with SSRP1 from low-salt and high-salt extracts but do not coimmunoprecipitate in the pellet fraction. The immunoprecipitations shown were performed on the same U2OS extracts. Western blots for the low-salt immunoprecipitations are shown on a separate gel.

To gain molecular insight into the mechanism of CENP-T/-W deposition, we set out to identify putative binding partners of CENP-T/-W. We followed a method previously used to successfully identify CenH3^CENP-A^ and H3–H4 histone chaperones (Tagami et al. 2004; Dunleavy et al. 2009). We used a HeLa cell line expressing GFP-tagged CENP-W in which GFP-tagged CENP-W localizes at centromeres as previously described (Fig. 2A; Hori et al. 2008). We then derived cell extracts (Fig. 2B) using low-salt, high-salt, and pellet subcellular fractions that were enriched for cytosolic, nuclear, and chromatin-bound proteins, respectively (Martini et al. 1998; Cook et al. 2011), and isolated proteins interacting with either CENP-W-GFP or GFP alone in a GFP-Trap pull-down. After analysis by gel electrophoresis and silver stain (Fig. 2B), we carried out mass spectrometry (MS) in biological triplicates for both samples and controls on low-salt and high-salt extracts. This provided us with a set of putative CENP-W-interacting proteins (Fig. 2B; Supplemental Table S1). Our MS analysis identified CENP-T and the CCAN components CENP-C and CENP-H, previously reported in complexes with CENP-T/-W (Foltz et al. 2006; Basilico et al. 2014) in addition to NPM1 (Chun et al. 2011) and EZH2 (Koh et al. 2015). The CENP-S/-X centromere complex, reported to form a heterotetramer with CENP-T/-W (Nishino et al. 2012), was not identified as a CENP-W-GFP interactor by our MS analysis. This is consistent with recent reports that predict that CENP-T/-W and CENP-S/-X are part of separate protein cohorts within the kinetochore (Samejima et al. 2015). In agreement with previous reports regarding CENP-T/-W interactions, we did not find CenH3^CENP-A^ in our CENP-W-GFP pull-downs (Hori et al. 2008; Abendroth et al. 2015), confirming the specificity of our approach. The presence of both FACT chaperone complex subunits (SSRP1 and Spt16) in both the low-salt and high-salt sample fractions of our MS analysis caught our attention, especially given that FACT has previously been reported as a component of centromeric chromatin pull-downs where CENP-T (also annotated as ICEN22/ FLJ13111) was also found (Foltz et al. 2006; Izuta et al. 2006). The FACT complex's function as a H2A–H2B histone chaperone is well characterized (Belotserkovskaya et al. 2003). The known structural similarity between histone dimers and the CENP-T/-W complex (Hori et al. 2008; Nishino et al. 2012) prompted us to ask whether FACT could also act as a binding partner for CENP-T/-W. To explore further whether FACT and CENP-T/-W could form a complex, we used antibodies specific for SSRP1 (Orphanides et al. 1999) and performed a combination of reciprocal coimmunoprecipitation (co-IP) experiments. We used extracts from cells expressing CENP-W-GFP (Supplemental Fig. S3a) and found that Spt16, CENP-T, and CENP-W coimmunoprecipitate with SSRP1 (Supplemental Fig. S3b). We next decided to explore further how FACT binds to CENP-T/-W. To delineate the composition of complexes containing FACT and CENP-T/-W, we tested cytosolic, nuclear, and chromatin-enriched cellular fractions using an untagged cell line (Supplemental Fig. S3c). We found SSRP1 and Spt16 in all subcellular fractions and thus decided to perform reciprocal SSRP1 co-IPs from each fraction. Spt16 coimmunoprecipitates with SSRP1 from all subcellular fractions (Fig. 2C). We found that CENP-T and CENP-W also coimmunoprecipitate with SSRP1 from the low-salt extract and the high-salt fraction. However, CENP-T/-W did not coimmunoprecipitate with SSRP1 in the chromatin-enriched pellet fraction (Fig. 2C). Given that CENP-T/-W protein levels peak in S phase (Prendergast et al. 2011), we prepared extracts from both asynchronous and S-phase-synchronized populations (Supplemental Fig. S3d). Again, we found that Spt16 and CENP-T/-W coimmunoprecipitate with SSRP1 from the low-salt extract and the high-salt fraction in both asynchronous and synchronized populations (Supplemental Fig. S3e). In agreement with our MS results, we did not detect the histone fold proteins CENP-A, CENP-S, or H2A coimmunoprecipitating with FACT from low-salt or high-salt extracts, suggesting that the FACT–CENP-T/-W interaction detected in these extracts is specific (Fig. 2C). Interestingly, CENP-C coimmunoprecipitates with SSRP1 in the chromatin fraction (pellet) in the absence of CENP-T/-W. However, we did not find CENP-C coimmunoprecipitating with FACT–CENP-T/-W in the low-salt or high-salt extracts. This suggests that the FACT–CENP-T/-W complex is distinct from the CCAN engagement of CENP-T/-W. Taken together, these data suggest that FACT forms a complex with CENP-T/-W in soluble form and when it is loosely associated with chromatin. Thus, our data indicate that FACT could act as a significant factor in the transfer and dynamics of soluble CENP-T/-W.

### The Spt16 C-terminal domain (Spt16-CTD) is sufficient to bind CENP-T/-W in vitro

To test whether FACT and CENP-T/-W interact directly, we used recombinant proteins to perform in vitro binding assays. We expressed tagged SSRP1-6xHis and Flag-Spt16 individually in insect cells for affinity purification, as previously described (Orphanides et al. 1999). We also coexpressed and purified a region of human CENP-T containing the HFD from amino acids 431–565 (CENP-T^HFD^) with full-length untagged CENP-W (Supplemental Fig. S4a). We then performed in vitro binding assays between CENP-T^HFD^/-W and either recombinant SSRP1-6xHis or Flag-Spt16. We found that both SSRP1-6xHis and Flag-Spt16 could independently bind to the CENP-T-^HFD^/-W complex (Supplemental Fig. S4b). These data demonstrate that the histone fold region of CENP-T with CENP-W can bind directly to both subunits of FACT.

We further explored the interaction between SSRP1 and CENP-T/-W. SSRP1 contains five well-characterized domains (Supplemental Fig. S4a; Winkler and Luger 2011; McCullough et al. 2013). Using expressed and purified recombinant GST-tagged truncation mutants of SSRP1, we found that only those that contained the acidic intrinsic disordered domain (IDD) of SSRP1 (amino acids 432–514) bound to the CENP-T^HFD^/-W complex (Supplemental Fig. S4c). The SSRP1-IDD and CENP-T^HFD^/-W interaction did not persist under high-salt conditions (data not shown), and, when subjected to gel filtration chromatography, they did not coelute (Supplemental Fig. S4d). These data suggested to us that the SSRP1-IDD and CENP-T^HFD^/-W may not form a stable complex.

**Figure 3.**
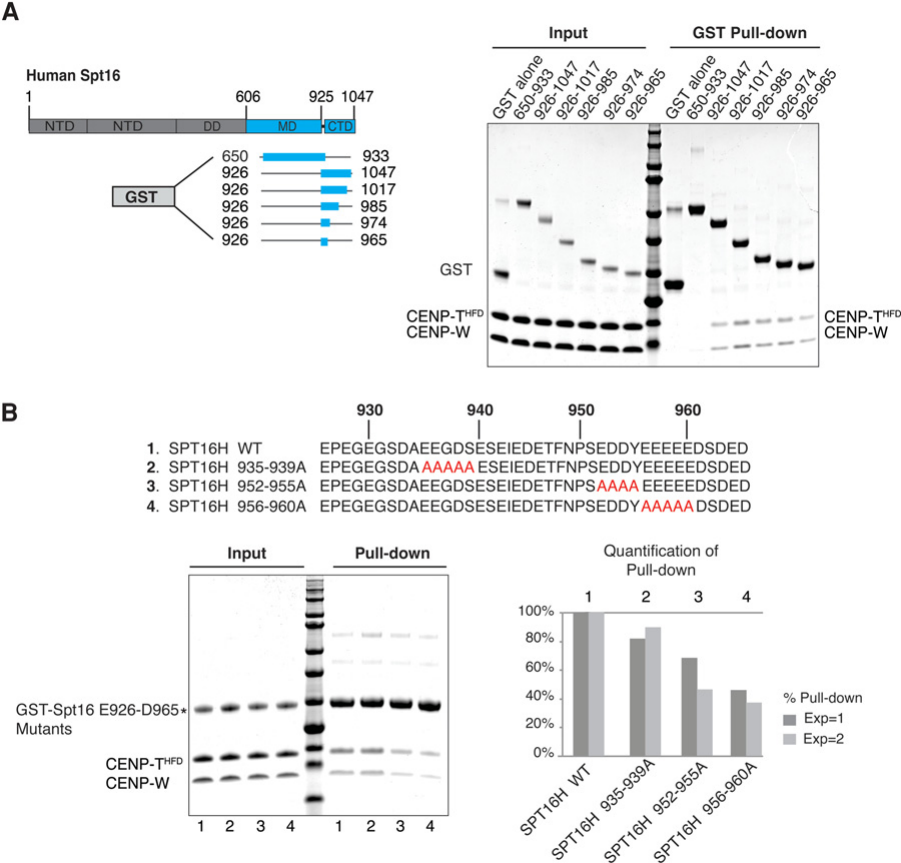
The Spt16-CTD is sufficient to bind CENP-T/-W in vitro. (*A*) Scheme depicting domains of Spt16 (amino acids within the respective domains summarized in Winkler and Luger 2011). The regions of Spt16 that were expressed as GST-tagged recombinant proteins are illustrated. Recombinant GST-tagged Spt16 fragments were used as bait in a GST pull-down assay. CENP-T^HFD^/-W was used as prey. GST alone was used as a control for pull-downs. Mutants that contained Spt16 amino acids 926–965 could bind CENP-T^HFD^/-W. (*B*) Schematic illustrating the amino acids within Spt16 (amino acids 926–965), which were mutated to alanine and expressed as recombinant GST-tagged proteins. The Coomassie gel shows input samples and GST pull-downs using GST-tagged Spt16 mutants as bait, and CENP-T^HFD^/-W was used as prey. GST alone was used as a pull-down control. The bar chart shows quantification of the amount of CENP-T/-W pulled down by each Spt16 mutant in two independent experiments.

To understand how Spt16 interacts with CENP-T-/W, we next tested which domains of Spt16 could bind to CENP-T^HFD^/-W. Spt16 contains several well-characterized functional domains (Fig. 3A; Winkler and Luger 2011; McCullough et al. 2013). We expressed GST-tagged truncated forms of Spt16 that spanned the middle and C-terminal acidic domains (Fig. 3A). We found that an acidic patch of Spt16 (amino acids 650–933) did not bind to CENP-T^HFD^/-W. In contrast, we observed that Spt16 amino acids 926–1047 could bind to CENP-T^HFD^/-W (Fig. 3A). This interaction was largely resistant to high salt (1 M NaCl) (Supplemental Fig. S5a). Furthermore, when we combined Spt16 amino acids 926–1047 and the CENP-T^HFD^/-W complex and subsequently subjected them to gel filtration chromatography, we found that they coeluted (Supplemental Fig. S5b), suggesting that they can form a stable complex. To further refine the region of the Spt16-CTD that could bind to CENP-T^HFD^/-W, we generated additional shorter truncated forms of Spt16. All truncated forms of Spt16 containing amino acids 926–965 bind CENP-T^HFD^/-W (Fig. 3A), and thus we conclude that an ∼39-amino-acid region of the CTD region of Spt16 (amino acids 926–965) is sufficient to bind to CENP-T^HFD^/-W. Taken together, our data indicate that FACT forms a stable direct interaction with CENP-T/-W via its subunit, SPT16, between amino acids 926 and 965. To further characterize the predicted CENP-T/-W-binding domain of Spt16, we mutated stretches of amino acid residues within the Spt16-CTD (amino acids 926–965) to alanine. We then tested the interaction between the GST-tagged Spt16 mutants and CENP-T^HFD^/-W in a GST pull-down assay. We found that mutation of residues amino acids 935–939 to alanine (Spt16 935–939A) had no significant effect on the binding to CENP-T^HFD^/-W (Fig. 3B). However, mutating amino acids 952–955 to alanine (Spt16 952–955A) reduced the binding to CENP-T^HFD^/-W by up to 40%, while mutating amino acids 956–960 to alanine (Spt16 956–960A) reduced the binding by up to 60%. Thus, our truncation analysis revealed key regions within the CTD of Spt16 that are critical for efficient binding of CENP-T^HFD^/-W.

### Spt16 can bind either H2A–H2B or CENP-T/-W

**Figure 4.**
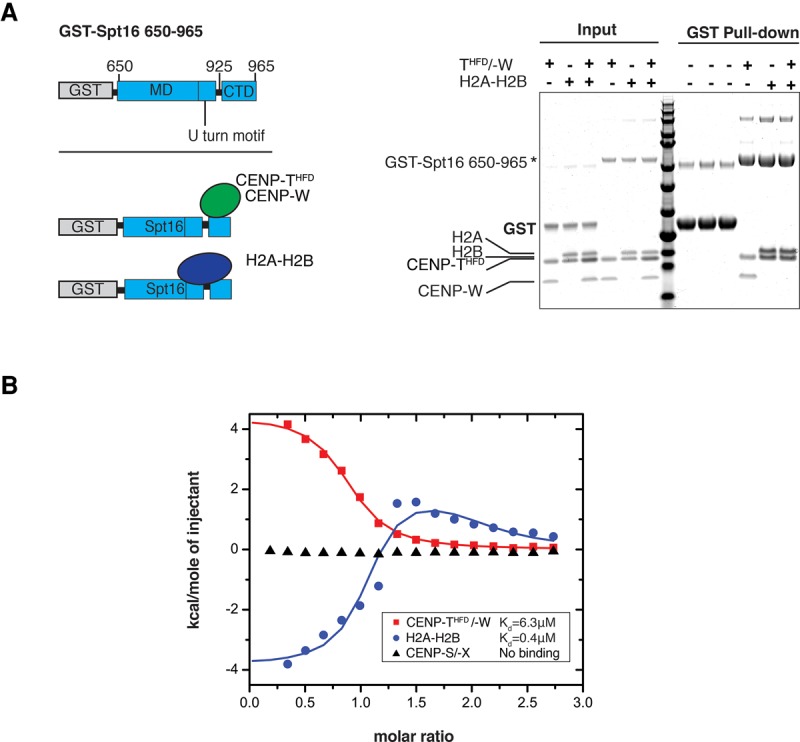
H2A–H2B and CENP-T^HFD^/-W bind to Spt16. (*A*) The schematic shows the region of Spt16 expressed as a GST fusion protein. The GST pull-down assay was performed using GST-Spt16 (amino acids 650–1047) to pull down H2A–H2B and/or CENP-T^HFD^/-W. The Coomassie gel shows input samples and GST pull-downs. GST-Spt16 (amino acids 650–1047) was combined in a 1:2 molar ratio with either CENP-T^HFD^/-W, H2A–H2B, or CENP-T^HFD^/-W and H2A–H2B combined. GST alone was used as a pull-down control. (*B*) Isothermal titration calorimetry (ITC) measurements for the Spt16-CTD with CENP-T/-W, H2A–H2B, and CENP-S/-X. The binding of the Spt16-CTD with CENP T/-W is endothermic, with a *K*_d_ value of 6.3 µM. There are two transitions for the binding of the Spt16-CTD with H2A–H2B, with a *K*_d_1 estimated at 0.4 µM and a *K*_d_2 estimated at 17 µM. There is no interaction between the Spt16-CTD and the centromeric histone fold proteins CENP-S/-X. The curve of CENP T/W was fit to a single-site binding model, and the curve of H2A–H2B was fitted to a two-site binding model.

FACT has been described as a H2A–H2B chaperone (Orphanides et al. 1999). In early studies on complexes involving fused constructs, it was proposed that the Spt16:H2A–H2B interaction region involved primarily the Spt16-MD U-turn and H2Bα1 helix, with the Spt16-CTD also contributing exothermically to the overall interaction with H2A–H2B (Fig. 4A; Hondele et al. 2013). Recent structural analyses of the *S. cerevisiae* Spt16-CTD:H2A–H2B complex has challenged the role of the Spt16-MD U-turn in targeting H2A–H2B and instead revealed that the primary H2A–H2B-binding site exists within the acidic Spt16-C and Pob3-C (SSRP1) domains (Kemble et al. 2015), a conclusion independently validated by others (Tsunaka et al. 2016). Interestingly, the minimal region of Spt16 that binds H2A–H2B (S965–E990 in *S. cerevisiae*) (Kemble et al. 2015) corresponds to approximately the same region of human Spt16 (Spt16 956–960), which we identified to bind to CENP-T/-W. Therefore, we asked whether human Spt16 simultaneously binds to both CENP-T/-W and H2A–H2B. We expressed and purified recombinant H2A–H2B and recombinant Spt16 amino acids 650–1047, which encompass the predicted H2A–H2B-binding and CENP-T/-W-binding domains. We then performed in vitro binding experiments in which H2A–H2B and/or CENP-T^HFD^/-W were incubated with GST-Spt16 amino acids 650–1047, and the resulting complexes were recovered by GST pull-down (Fig. 4A). In agreement with previous data (Hondele et al. 2013; Kemble et al. 2015), we confirmed that Spt16 amino acids 650–1047 binds to H2A–H2B. We then confirmed that that Spt16 amino acids 650–1047 binds to CENP-T^HFD^/-W (Fig. 4A). However, when equimolar amounts of CENP-T^HFD^/-W and H2A–H2B were combined, only H2A–H2B bound to Spt16 (Fig. 4A). These data suggest that Spt16 does not bind both partners simultaneously.

We next wanted to provide insight into the mechanism through which Spt16 may handle different binding partners. We performed isothermal titration calorimetry (ITC) to measure the binding affinities between the Spt16-CTD (Spt16 amino acids 926–965) and three separate HFD-containing complexes (H2A–H2B, CENP-T/-W, and CENP-S/-X) (Fig. 4B). CENP-S/-X is a histone fold complex structurally similar to both CENP-T/W and H2A–H2B and is reported to form a heterotetrameric complex with CENP-T/-W in vitro (Nishino et al. 2012). We found that the binding of the Spt16-CTD with CENP-T/-W is endothermic, with a *K*_d_ value of 6.3 µM. This binding is consistent with there being only one site on CENP-T/-W for binding with the Spt16-CTD. There are two transitions for the binding of the Spt16-CTD with H2A–H2B, involving, namely, an exothermic primary high-affinity binding event with *K*_d_1 = 0.4 µM affinity and an endothermic secondary low-affinity binding event with *K*_d_2 = 17 µM affinity. Notably, the binding of the Spt16-CTD to H2A–H2B occurs by an exothermic process (primary binding event, *K*_d_ = 0.4 µM), while binding to CENP-T^HFD^/-W occurs by an endothermic process (*K*_d_ = 6.3 µM). There is no straightforward explanation for this difference, but it could reflect distinct contributions from the difference in sequence of the Spt16-CTD-binding sites on H2A–H2B versus CENP-T^HFD^/-W. It should be noted that the interactions of the human Spt16-CTD and human H2A–H2B are more extensive than the interactions observed for their counterparts in *S. cerevisiae* (Kemble et al. 2015; Y Liu and DJ Patel, unpubl.). There is no interaction detected between the Spt16-CTD and the CENP-S/-X complex (Fig. 4B). Together, these data show that the Spt16-CTD exhibits higher affinity for H2A–H2B than for CENP-T/-W and that the interaction of the Spt16-CTD with both H2A–H2B and CENP-T/-W is specific.

### FACT regulates in vivo dynamics of CENP-T/-W

**Figure 5.**
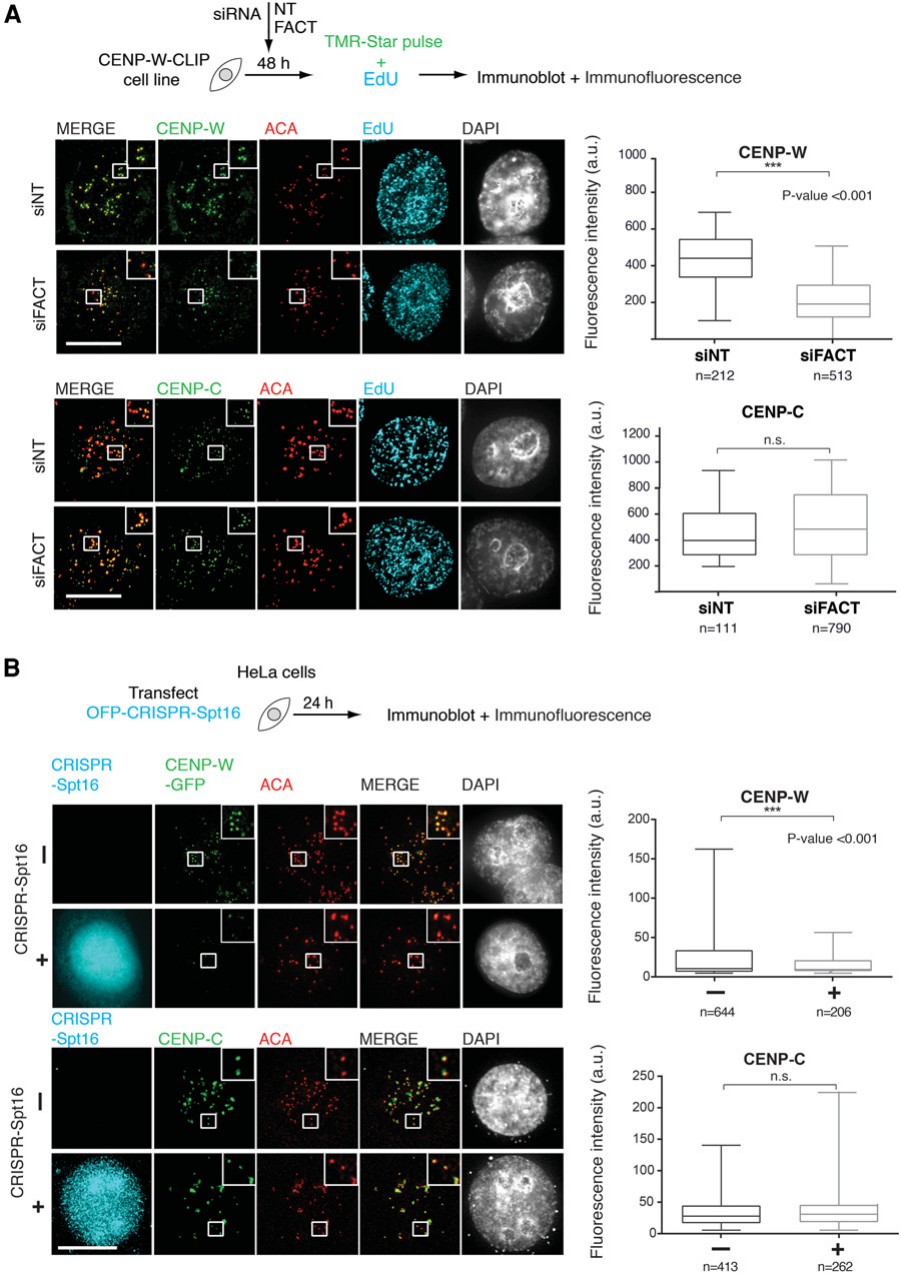
FACT or Spt16 depletion impairs centromeric deposition of CENP-T/-W. (*A*) Experimental scheme outlining the approach. HeLa cells stably expressing CENP-W-CLIP were depleted of FACT for 48 h by siRNA. The levels of proteins of interest were assayed by Western blot (Supplemental Fig. S6a). EdU incorporation was used as a marker of S phase. CENP-W-CLIP was visualized by labeling with CLIP cell TMR-Star. ACA signal was used to identify centromeres. Experiments were repeated three times. A minimum of 30 cells was quantified per experiment. *n* = number of cells quantified. The plot shown is a scatter plot and includes all values for one representative experiment. *P*-values were calculated using a two-tailed student *t*-test with Welch's correction. Maximum intensity projections were used to prepare images. Bar, 10 µM. (*B*) The schematic outlines the CRISPR–Cas9 approach used to disrupt Spt16. The experimental scheme outlines the approach. Custom CRISPR–Cas9–Spt16 targeting construct (CRISPR–Spt16) plasmids carrying an orange fluorescent protein (OFP) reporter were transfected into cells stably expressing CENP-W-GFP or wild-type HeLa cells. Centromere intensities of CENP-W-GFP, CENP-T, and CENP-C were quantified in both cells expressing CRISPR–Spt16 and untransfected cells. ACA was used to identify centromeres. Quantification was performed in three dimensions on deconvolved *Z* stacks using an automated ImageJ macro (see the Supplemental Material). Experiments were repeated four times. The graphs shown are scatter plots of all centromere intensities. A minimum of 30 cells was quantified per experiment. *n* = number of cells quantified. *P*-values for significance were calculated using a two-tailed student *t*-test with Welch's correction. Maximum intensity projections were used to prepare images. Bar, 10 µM.

Our data indicate that CENP-T/-W interacts directly with FACT prior to their deposition to centromeres. We therefore tested the functional importance of this interaction for the centromere localization of CENP-T/-W. We first used siRNA against SSRP1 and Spt16 to deplete FACT in a cell line stably expressing exogenous CLIP-tagged CENP-W, allowing us to visualize CENP-W (Supplemental Fig. S6a). After 48 h, centromeric CENP-W-CLIP levels were significantly reduced (Fig. 5A), while the levels of centromeric CENP-C were not affected. These data indicate that loss of FACT significantly reduces centromeric accumulation of CENP-W.

Using an alternative depletion strategy, we next tested whether loss of Spt16 affected the deposition of CENP-T, CENP-W, or CENP-C to centromeres. To specifically deplete Spt16 from cells, we exploited the CRISPR–Cas9 genome-editing system (Cong et al. 2013; Mali et al. 2013). CRISPR–Cas9 has been implemented successfully as an alternative to siRNA in high-throughput screening approaches to knock out a broad spectrum of mammalian genes within, importantly, a short time window (Shalem et al. 2014; Wang et al. 2014; Zhou et al. 2014). Deletion of amino acids 816–1047 in the Spt16-CTD causes loss of Spt16 chaperone function (Belotserkovskaya et al. 2003). We designed custom CRISPR–Cas9–Spt16 targeting constructs (CRISPR–Spt16) carrying an orange fluorescent protein (OFP) reporter cassette engineered to cut on either side of Spt16 exons 24–26. This approach excises the region from amino acids 926–1047 in vivo (Fig. 5A; Supplemental Fig. S6b). Immunofluorescence using Spt16 antibodies showed that cells that were transiently transfected with CRISPR plasmids were depleted for Spt16 within 24 h (Supplemental Fig. S6b,c). The depletion of total Spt16 protein levels in the population (where ∼20%–30% of cells were transfected) as assayed by Western blot was ∼30% (Supplemental Fig. S6d). This is consistent with the level of CRISPR–Spt16 transfection efficiency, as only targeted cells lose Spt16. We did not detect the appearance of a truncated Spt16 protein by Western blot. To observe the effects of Spt16 loss in vivo, we transfected cells with CRISPR–Spt16 and performed live-cell imaging. We observed a high rate of cell death in transfected cells within 24 h, as compared with untransfected cells (Supplemental Fig. S6e; Supplemental Movie S1). This is in agreement with the essential biological role of FACT, with loss of SSRP1 being embryonic-lethal (Cao et al. 2003). For this reason, we used a transient transfection approach for our analyses. Similarly to the siRNA approach, we found that CENP-W-GFP cells expressing CRISPR–Spt16 had both reduced Spt16 and significantly reduced CENP-W-GFP at centromeres (Fig. 5B), while endogenous centromeric CENP-C signals in CRISPR–Spt16-positive cells remained unaffected (Fig. 5B). A significant reduction in CENP-T levels at centromeres was also apparent (Supplemental Fig. S6f). This indicates that the reduction observed in CENP-W and CENP-T centromeric levels following loss of FACT is not an indirect consequence of CENP-C loss and suggests that FACT may contribute to CENP-T/-W dynamics in vivo.

### Spt16 is sufficient to promote local accumulation of CENP-T

**Figure 6.**
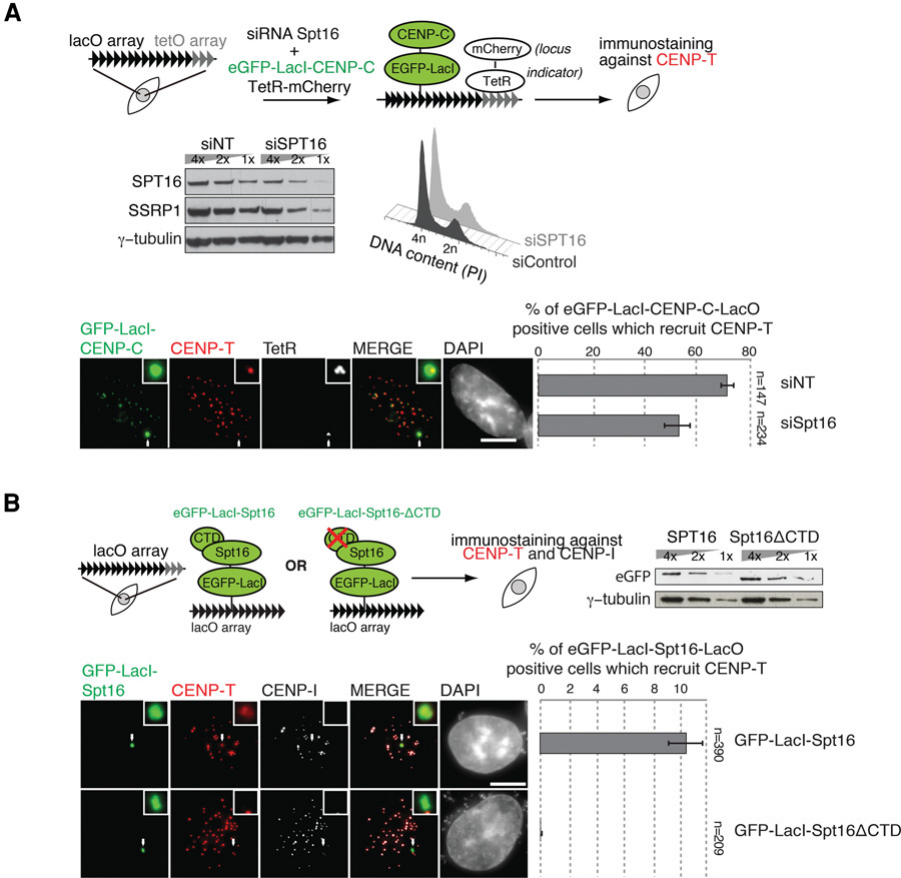
Spt16 is sufficient for de novo CENP-T accumulation. (*A*) The schematic outlines the LacO–LacI-tethering approach. A cell line stably expressing a LacO-TetR array was transfected with siRNA (siNT or siSpt16) for 24 h. The levels of proteins of interest were assayed by Western blot. FACS analyses show cell cycle profiles of the treated samples. Following 24 h, siRNA-treated cells were transiently transfected with eGFP-LacI-CENP-C and TetO-mCherry. Cells were then fixed after a further 24 h, and immunofluorescence for CENP-T was performed. TetR-mCherry allows visualization of the LacO-tethered eGFP-LacI-CENP-C. The percentage of eGFP-LacI-CENP-C-positive cells in which CENP-T was recruited by tethered CENP-C was counted. In siSpt16-treated samples, CENP-T recruitment was impaired. (*B*) The schematic outlines the LacO–LacI-tethering approach. A cell line stably expressing a LacO-TetR array was transiently transfected with eGFP-LacI-Spt16 or a truncated Spt16 (eGFP-LacI-Spt16ΔCTD) missing Spt16 amino acids 940–1047. Following 24 h, cells were fixed, and immunofluorescence for CENP-T and CENP-I was performed. The percentage of eGFP-LacI-Spt16-positive cells in which CENP-T colocalized with either eGFP-LacI-Spt16 (*n* = 390) or eGFP-LacI-Spt16ΔCTD (*n* = 209) was counted. CENP-T was not recruited by eGFP-LacI-Spt16ΔCTD.

Since our observations could be a result of indirect effects due to the role of FACT in transcription, we wished to provide more direct evidence for a role for FACT in de novo CENP-T/-W accumulation. For this, we used the LacO–LacI system that we used previously, where we showed that tethered LacI-CENP-C at a LacO DNA array recruits CENP-T to the locus (Tachiwana et al. 2015). Here, we used this system in order to assess whether Spt16 was involved in de novo accumulation of CENP-T at a LacO array tethered with eGFP-LacI-CENP-C. Approximately 74% of eGFP-LacI-CENP-C transfected cells recruited CENP-T to the LacO locus, as described previously (Fig. 6A; Supplemental Fig. S7; Tachiwana et al. 2015). siRNA targeting Spt16 reduced the accumulation of CENP-T to the locus tethered with eGFP-LacI-CENP-C by ∼25% within 24 h (Fig. 6A). These data indicate that Spt16 is required for the efficient de novo accumulation of CENP-T to a locus tethered with eGFP-LacI-CENP-C. Since FACT is a pleiotropic factor with many roles in cellular metabolism, its disruption may indirectly impact centromere function. We thus tested whether a direct artificial tethering of Spt16 to DNA using the LacI–LacO system could stimulate de novo local CENP-T accumulation. We transfected eGFP-LacI-Spt16 into cells that stably harbor the LacO array. After 24 h, we performed immunofluorescence for endogenous CENP-T. The CCAN component CENP-I was used as an endogenous kinetochore marker as a control to ensure that we could efficiently identify the LacO locus. We found that CENP-T accumulates at the LacO locus in ∼10% of cells transfected with eGFP-LacI-Spt16 (Fig. 6B). We performed the same experiment using a truncated Spt16 lacking the region of the CTD identified to be important for CENP-T/-W binding (amino acids 940–1047 [LacI-eGFP-Spt16ΔCTD]). The truncated LacI-eGFP-Spt16 fusion was stable and expressed at a level to similar that of the full-length construct (Fig. 6B). However, CENP-T was not recruited to the locus. Taken together, these data show that tethering Spt16 to the LacO locus is sufficient to stimulate CENP-T accumulation in vivo and that CENP-T accumulation in vivo depends on the CTD of Spt16.

## Discussion

CENP-T/-W deposition at centromeres occurs de novo during S phase (Prendergast et al. 2011), and our data confirm this finding. However, we found that CENP-T deposition at centromeres is uncoupled from DNA synthesis (Fig. 1). This suggests that events occurring in S phase, other than DNA synthesis, may promote CENP-T/-W deposition. RNA Pol II transcription at centromeres is promoted by modification of centromeric H2B (Sadeghi et al. 2014) and has been suggested to promote centromere organization (Bergmann et al. 2011; Choi et al. 2011). Furthermore, long noncoding centromeric transcripts, also localizing to centromeres, play a role in the localization of CENP-A in human cells (Quénet and Dalal 2014) and CENP-C and CENP-A in flies (Rošić et al. 2014). Since transcription may be necessary for centromere function (for review, see Bergmann et al. 2012; Scott 2013), we wondered whether this was true for CENP-T/-W deposition. Here, we observed that DRB inhibition of RNA Pol II Ser2 phosphorylation reduces CENP-T and CENP-W accumulation at centromeres. As the synthesis of “new” CENP-T-CLIP and CENP-W-CLIP is not significantly reduced in this case, it is unlikely that the observed decrease in centromere deposition is simply due to impaired protein synthesis. However, these data do not allow us to determine whether RNA Pol II transcription is directly or indirectly involved in CENP-T/-W deposition at centromeres. This led us to explore possible connections between CENP-T/-W and other factors that could act to regulate CENP-T/-W dynamics.

Our analysis of CENP-T/-W candidate binding partners (Fig. 2B) revealed FACT as a functional player in CENP-T/-W dynamics. Specifically, our work identified a novel role for the FACT subunit Spt16 in binding to the CENP-T/-W complex and regulating the accumulation of CENP-T/-W to centromeres. We also found interactions between SSRP1 and CENP-T/-W, albeit less stable than those observed for Spt16 and CENP-T/-W. Given that either CTD of *S. cerevisiae* SSRP1(Pob3) or Spt16 can bind to H2A–H2B (Kemble et al. 2015), we cannot exclude a role for SSRP1 in CENP-T/-W binding in vivo. Here, we demonstrate that Spt16 binds to CENP-T/-W through a 39-amino-acid region of the Spt16 CTD (Fig. 3), thereby forming a stable complex. The region of Spt16 that is sufficient to bind CENP-T/-W (Spt16 amino acids 956–960) is within the minimum region of *S. cerevisiae* Spt16 (amino acids 958–999), determined to be sufficient to bind H2A–H2B (Kemble et al. 2015). No H2A–H2B binding by the Spt16MD domain was detected with either yeast or human Spt16 (Kemble et al. 2015; Tsunaka et al. 2016), and, similarly, we did not find any binding between the human Spt16MD domain (amino acids 650–933) and CENP- T^HFD^/-W. The interaction between CENP-T/-W and FACT excludes the interaction between FACT and H2A–H2B and exhibits distinct properties. First, we show that FACT binds to CENP-T/-W in low-salt cytosolic extracts, where FACT bound to H2A is not detected (Fig. 2C). Second, we show that H2A–H2B has a higher affinity for Spt16 than CENP-T/-W (Fig. 4B). The ITC data indicate that the binding of the Spt16-CTD to the only site on CENP-T/-W has characteristics similar to the binding to the secondary site on H2A–H2B, as both are endothermic. Alternatively, the location of the binding site on CENP-T/-W could be similar to the primary binding site on H2A–H2B, but different amino acids on the binding interfaces of CENP-T/-W could contribute to the different thermodynamic properties of interactions. Given the higher affinity of Spt16 for H2A–H2B (∼15-fold higher), we speculate that free H2A–H2B may be a negative regulator of the FACT–CENP-T/-W complex. Therefore, the interactions between FACT and CENP-T/-W, which we detected by immunoprecipitation (Fig. 2; Supplemental Fig. S4), may occur in the presence of a limited amount of free H2A–H2B; for example, when soluble H2A–H2B is sequestered by a dedicated chaperone such as Nap1 (Mosammaparast et al. 2002) or during replication of centromeric DNA late in S phase when free H2A–H2B becomes limited.

The association of FACT with CENP-C in the chromatin fraction raises the possibility that centromeric CENP-C may assist correct targeting of CENP-T/-W. This could be mediated by the CENP-H/-I/-K/-M complex, which has a demonstrated role in recruiting CENP-T to centromeres downstream from CENP-C (Basilico et al. 2014). Indeed, interactions between FACT and CENP-H have been reported (Okada et al. 2009). In support of this, our protein interaction assays using extracts and purified proteins show that FACT can form a complex with CENP-T/-W and CENP-C, albeit in different biochemical fractions (Fig. 2C). We show that the loss of FACT through either siRNA or CRISPR depletion of Spt16 reduces the levels of CENP-W and CENP-T, but not CENP-C, at endogenous centromeres (Fig. 5A). Furthermore, we show that depletion of Spt16 impacts the de novo recruitment of CENP-T by LacO-tethered CENP-C (Fig. 6A). Together, these data indicate that FACT is involved in regulating CENP-T/-W accumulation at centromeres downstream from CENP-C. Strikingly, we demonstrate that Spt16 alone tethered at a LacO locus promotes local de novo accumulation of CENP-T, supporting our conclusion that Spt16 and CENP-T can interact in vivo (Fig. 6B). Furthermore, our structure–function analyses demonstrate that the region of the Spt16 CTD (amino acids 940–stop) that binds CENP-T/-W in vitro (Fig. 3B) is necessary for CENP-T accumulation at GFP-LacI-Spt16 tethered at LacO (Fig. 6B). This underscores the functional significance of the interaction between CENP-T/-W and Spt16 and further supports a role for Spt16 as a binding partner of CENP-T/-W.

Overall, the interaction that we describe between Spt16 and CENP-T/-W suggests that, similarly to chaperone–histone complexes, FACT may function to stabilize the soluble CENP-T/-W complex in the cell and facilitate its nuclear dynamics. First, the CENP-T/-W complex is structurally homologous to the histone H2A–H2B dimer (Hori et al. 2008; Nishino et al. 2012), and we found that it could bind to Spt16 in a similar region (Fig. 3). Second, while CENP-T/-W is resident at mitotic kinetochores, Spt16 is not, which indicates that FACT does not function as an architectural component at centromeres to anchor CENP-T/-W. This is also in line with our observation that the complex is found in the extractable fraction and not in the chromatin pellet (Fig. 2C). Considering the definition of a histone chaperone as a factor that participates in histone transfer in reactions without being part of the final product, Spt16 fulfills criteria that make it eligible as a bona fide CENP-T/-W chaperone. How FACT would facilitate targeting of CENP-T/-W to centromeres specifically is an open question. We show that Spt16 directly binds to the HFDs of the CENP-T/-W complex (Fig. 3). The importance of the CENP-T HFD for centromere localization of the protein has previously been demonstrated. CENP-T lacking the CENP-T α4 and α5 helices fails to localize to kinetochores in HeLa cells (Nishino et al. 2012). Phosphorylation of CENP-T within the N-terminal region (Nishino et al. 2013) and C-terminal region (Gascoigne and Cheeseman 2013) regulates CENP-T centromere localization. Therefore, one scenario could involve cell cycle-regulated phosphorylation of CENP-T at either the N-terminal or C-terminal region acting to guide the FACT–CENP-T/-W complex to centromeres. An alternative possibility is the in vivo regulation of the local availability of CENP-T/-W to Spt16, which could boost the opportunities for interaction. Similarly, in vivo regulation of the binding between Spt16–CENP-T/-W or Spt16–H2A–H2B could play a functional role in the interactions. At this point, it would be interesting to delineate whether there can be competition between the Spt16-binding partners. Regardless of whether there is competition, it is tempting to speculate that an exchange between CENP-T/-W and H2A–H2B could participate in the regulation or coordination of a delivery mechanism at centromeres (Supplemental Fig. S8). In this scenario, the delivery of CENP-T/-W would be coordinated with the removal of H2A–H2B from centromeric chromatin. For example, when FACT is recruited to chromatin (in the context of transcription or concomitant with replication), there is disruption or mobilization of H2A–H2B from chromatin. As attested by the ITC data, the higher affinity of Spt16 for H2A–H2B could promote the exchange or release of CENP-T/-W. If this happens outside centric regions, no stable chromatin incorporation of CENP-T/-W may occur due to the absence of retention mechanisms. However, when encountering centric regions, CENP-T/-W may readily bind to centromeric CENP-T/-W already present or bind the CENP-H/-I/-K/-M complex recruited by CENP-C (Basilico et al. 2014; Klare et al. 2015; McKinley et al. 2015). This retention could ultimately result in CENP-T/-W accumulation. In addition, the late replication time of the centromeric regions should coincide with a relative exhaustion of the soluble pool of H2A–H2B, thus also favoring the choice of CENP-T/-W to be loaded.

FACT is a histone chaperone with known cellular functions in DNA replication, repair, and transcription (Winkler and Luger 2011; Winkler et al. 2011). While several studies have proposed an important role for FACT in centromere function, our data provide the first direct molecular link between FACT and centromere components. Through characterizing a new partner for FACT, we identified a novel pathway impacting mitotic function through regulating the dynamics of the CENP-T/-W complex. It will be important to address in future work how FACT would facilitate CENP-T/-W accumulation preferentially at centromeres. Indeed, whether CENP-T/-W is localized exclusively at centromeres or has functions at other genomic loci is unknown. Future work should aim to address whether additional factors regulate either the stability of FACT–partner interactions or CENP-T/-W, thus controlling the targeting and timing of CENP-T/-W accumulation.

## Materials and methods

### Purification of the CENP-W-GFP complex

We purified CENP-W-GFP complexes as described (Tagami et al. 2004; Dunleavy et al. 2009) and prepared nuclear extracts as described (Martini et al. 1998; Cook et al. 2011) with the addition of protease inhibitors B-glycerophosphate (10 mM), NaF (5 mM), and Na_3_VO_4_ (0.2 mM). After pull-down with GFP-Trap GFP-coupled sepharose (Chromotek) in immunoprecipitation buffer (300 mM NaCl), bound polypeptides were eluted in LDS sample buffer and identified by MS.

### Liquid chromatography-tandem MS (LC-MS/MS) analyses

Samples were analyzed by nano-LC-MS/MS using an RSLCnano system (Ultimate 3000, Thermo Scientific) coupled to an Orbitrap Fusion mass spectrometer (Q-OT-qIT, Thermo Fisher Scientific). For details of the separation, mass spectrometer parameters, and data analysis, see the Supplemental Material. The MS proteomics data have been deposited to the ProteomeXchange Consortium via the Proteomics Identifications (PRIDE) (Vizcaíno et al. 2016) partner repository with the data set identifier PXD004099.

### ITC experiments

All of the ITC titrations were performed on a Microcal ITC 200 calorimeter at 20°C. The exothermic heat of the reaction was measured by 17 sequential 2.2-L injections of the Spt16-CTD (amino acids 926–965; 2.2 mM in buffer, 20 mM Tris at pH 7.5, 0.2 M NaCl) into 200 µL of the CENP-T/-W, human H2A/H2B, and CENP S/X solution, respectively (150 µM in the same buffer), spaced at intervals of 150 sec. The data were processed with Microcal Origin software, the curve of CENP-T/-W was fit to a single-site binding model, and the curve of H2A/H2B was fitted to a two-site binding model.

### CLIP labeling

CLIP tag activity in cells was quenched by addition of 20 mM CLIP cell block (BTC) (New England Biolabs) in complete growth medium for 30 min at 37°C. CLIP-tagged proteins were pulse-labeled with 2 mM CLIP-Cell TMR-Star in complete medium supplemented with 1% BSA for 20 min at 37°C. Following quench or pulse labeling, cells were washed twice with prewarmed PBS and once with complete DMEM. Cells were then reincubated in complete medium for 20 min prior to fixation.

### CRISPR design and synthesis

CRISPR–Spt16 plasmids were custom-synthesized by Life Technologies. Custom specific Spt16 guide RNAs were subcloned to a vector carrying an OFP reporter and expressing Cas9. The guide RNA sequences used were Spt16_GuideRNA_F1 (GAGG GTGAGGTGCGTGAGTGTGG [sense]) and Spt16_Guide RNA_F2 (CTTCTTCAGCATCACTCCCCTGG [antisense]).

## Supplementary Material

Supplemental Material
