# Supplementary material for: The CENP-T/-W complex is a binding partner of the histone chaperone FACT
Source: Genes Dev. 2016 Jun 1;30(11):1313–26. doi: 10.1101/gad.275073.115 (PMC4911930; doi:10.1101/gad.275073.115)
Supplement: Supplemental Material [file supp_30_11_1313__index.html]

The CENP-T/-W complex is a binding partner of the histone chaperone FACT — Supplemental Material 

# The CENP-T/-W complex is a binding partner of the histone chaperone FACT

## Supplemental Material

**Files in this Data Supplement:**

- Supp\_Material.pdf
- Supplemental\_FigS4.pdf
- Supplemental\_FigS5.pdf
- Supplemental\_FigS6.pdf
- Supplemental\_FigS2.ai
- Supplemental\_MovieS1.avi
- Supplemental\_FigS3.ai
- Supplemental\_FigS8.ai
- Supplemental\_FigS1.ai
- Supplementary\_Figure\_Legends.docx
- Supplemental\_Table\_S1.xlsx
- Supplemental\_FigS7.ai
