# Supplementary material for: The CENP-T/-W complex is a binding partner of the histone chaperone FACT
Source: Genes Dev. 2016 Jun 1;30(11):1313–26. doi: 10.1101/gad.275073.115 (PMC4911930; doi:10.1101/gad.275073.115)
Supplement: Supplemental Material [file supp_gad.275073.115_Supp_Material.pdf]

Supplementary Materials and Methods

Mass spectrometry LC-MS/MS analysis of CENP-W-GFP and GFP control pull-downs

Following purification of CENP-W-GFP and GFP interacting factors, proteins were eluted in 1x LDS buffer (Life Technologies). Eluted proteins were separated by denaturing electrophoresis on SDS-polyacrylamide gels (Invitrogen). The gels were colored by colloidal blue staining (LabSafe GEL Blue GBiosciences) and 4 gel slices were excised, reduced, alkylated, and subjected to digestion with trypsin (Sequencing Grade, Promega) in 25 mM ammonium bicarbonate overnight at 30°C. The extracted peptides were analyzed by nano-LC-MS/MS using an RSLCnano system (Ultimate 3000, Thermo Scientific) coupled to an Orbitrap Fusion mass spectrometer (Q-OT-qIT, Thermo Fisher Scientific), Bremen, Germany). Samples are loaded on a C18 precolumn (300 µm inner diameter x 5 mm; Dionex) at 20 µl/min in 2% MeCN, 0.1% HCOOH. After 3 min of desalting, the precolumn was switched on line with the analytical C18 column (75 µm inner diameter x 25 cm; C18 PepMap^{TM}, Dionex) equilibrated in solvent A (5% MeCN, 0.1% HCOOH). Bound peptides were eluted using a linear gradient of 100 min (from 5 to 30% (v/v)) of solvent B (100% MeCN, 0.085% HCOOH) at a 300 nl/min flow rate and an oven temperature of 40°C. We acquired Survey MS scans in the Orbitrap on the 400-1500 m/z range with the resolution set to a value of 120,000 and a 4×10^5 ion count target. Each scan was recalibrated in real time by co-injecting an internal standard from ambient air into the C-trap. Tandem MS was performed by isolation at 1.6 Th with the quadrupole, HCD

fragmentation with normalized collision energy of 35, and rapid scan MS analysis in the ion trap. The MS2 ion count target was set to 10^4 and the max injection time was 100 ms. Only those precursors with charge state 2–7 were sampled for MS2. The dynamic exclusion duration was set to 60 s with a 10 ppm tolerance around the selected precursor and its isotopes. The instrument was run in top speed mode with 3 s cycles. Data were acquired using the Xcalibur software (v 3.0) and the resulting spectra were interrogated by Sequest HT through Proteome Discoverer (v 1.4.1.14, Thermo Scientific) using the *Homo sapiens* database (UniProtKB Human December 2015, 42086 sequences). Carbamidomethylation of cysteines, oxidation of Metionine, N-terminal acetylation were set as variable modifications for all searches. Specificity of trypsin digestion was set and two missed cleavage sites were allowed. Mass tolerances in MS and MS/MS were set to 10 ppm and 0.6 Da, respectively. The resulting files were further processed using *my*ProMS (Poullet et al. Proteomics 2007). The Sequest HT target and decoy search result were validated at 1% FDR with Percolator.

Cell Lines and Cloning

CENP-W stably expressed as a GFP fusion protein with a C-terminal GFP tag in HeLa S3 cells (Hori et al. 2008) was used for purification of CENP-W-GFP. A cell line stably expressing CENP-W as a CLIP tag fusion protein in HeLa cells was used as (Prendergast et al. 2011). Plasmids expressing 6xHis-SSRP1 and Spt16-FLAG were a gift from D. Reinberg.

Cell line harbouring a stable LacO array was a gift from E. Soutoglou. Plasmids for expressing eGFP-LacI fusions (Tachiwana et al. 2015) were adapted for use in with the Gateway recombination cloning system by L.P,

generating eGFP-LacI-GW. Spt16 and Spt16-CTD mutants were generated by PCR amplification from a plasmid containing full length Spt16 cDNA, generously provided by Prof. A. Ladurner. Plasmids eGFP-LacI-Spt16 and eGFP-LacI-Spt16-CTD were generated by recombination cloning to eGFP-LacI-GW.

Immunofluorescence microscopy and quantitation

We grew cells on glass coverslips and fixed cells either in 2% paraformaldehyde (PFA) for 20 min and permeabilised with 0.2% TritonX-100 for 5 min for 15 min at RT or fixed in PTMEF buffer (20 mM PIPES [pH 6.8], 4% PFA, 0.2% Triton X-100, 10 mM EGTA, 1 mM $MgCl_2$) for 20 minutes (Chan et al. 2009) and processed for IF. Coverslips were mounted in ProLong gold antifade reagent with DAPI (Invitrogen). Images were acquired on a DeltaVision Core system (Applied Precision) controlling an interline charge-coupled device camera (Coolsnap HQ2; Roper) mounted on an inverted microscope (IX- 71; Olympus). Images were collected at 1x1 binning using a 100x oil objective or 60x oil objective at 0.2 μm z sections. Images were deconvolved and maximum intensity projected using SoftWoRx (Applied Precision). For quantification, unscaled DeltaVision images were used. Centromere intensity was measured in 3D using ImageJ (adapted from (Cantaloube et al. 2012)). A centromere mask based on ACA or CENP-B signal was generated and applied to the target channel. Background was subtracted automatically using a Top-hat algorithm and mean intensity per centromere was calculated.

Western blot analysis

Protein extracts were either, fractionated and prepared as described above, or whole cell extracts for Western blot analysis were prepared by re-suspending cells in 1x LDS sample buffer (Life Technologies) supplemented with 10% DTT (Sigma) and benzonase. Samples were run on NuPAGE 4-12% gels (Invitrogen) in MES or MOPs buffer and transferred to a PVDF membrane or Nitrocellulose membranes (Protran, Germany). Membranes were stained with Memcode prior to blocking with 5% milk in PBS-Tween (0.1%). Primary and secondary antibodies were incubated with membranes in 5% milk in PBS-Tween (0.1%). Immunodetection was performed using Thermo Scientific™ SuperSignal™ West Pico or Femto chemiluminescent substrates. Western blot images were captured using an ImageQuant LAS-4000 system (GE healthcare) or exposure to film.

Immunoprecipitation

Immunoprecipitation (IP) experiments were carried out at 4°C for 16 h using 5 µg of antibody and 20 µl of sepharose to 500 µg of protein extract in IP buffer (20 mM Tris-HCL pH 7.5, 10% Glycerol, 300 mM NaCl, 0.1% NP40, 0.2 mM EDTA) + 0.1% BSA with Complete protease inhibitor cocktail (Roche) and additional protease inhibitors 10 mM B-Glycerophosphate, 5 mM NaF, 0.2 mM Na_3VO_4. The equivalent of 50% of the total IP was loaded per lane of an SDS-PAGE gel for analysis by coomassie or Western blot.

Antibodies

Anti-SSRP1 antibodies were gifts from D. Reinberg. Anti-Spt16 (rabbit, Santa Cruz, sc-28734, 1:500). Anti-CENP-T antibodies were a gift from I.M. Cheeseman. Anti-CENP-W (C6ORF173) antibody (rabbit, Abcam, ab189957, 1:500), Anti-CENP-S/MHF1 antibody (rabbit, Abcam, ab169385, 1:500), Anti-CENP-C (guinea pig, MBL international, PD030, 1:1000). Anti-GFP (rabbit, Santa Cruz, sc8334, 1:1000). Anti-RNA Pol II Ser2 (rat clone 3e10, Millipore, 04-1571, 1:1000) Anti-RNA Pol II Ser5 (rat, Millipore, 04-1572, 1:1000), Anti-RNA Pol II (8wg16) (rat, Millipore 1:1000). Gamma tubulin (mouse Sigma 1:5000). Anti-Centromere antibody (ENZO life sciences 1:1000).

RNAi and Cell Culture.

Cells were transiently transfected using JetPrime transfection reagent for siRNA and plasmid delivery. Plasmid transfections were also performed by electroporation using an AMAXA Nucleofector. siRNA (MWG Biotech) transfected cells were analyzed 48 h after transfection (double round of transfection).

siRNA sequences against FACT

SSRP1_si.5 GAAGAAGAACUAGCCAGUA,

SSRP1_si.2 AGAAUGGCCAUGUCUACAA,

SUPT16_si.5 AUAGUUGAUGCAGAUGAGA,

SUPT16_si.3 AUAGAUAUCAGUGCAGUUG

Supplemental references:

Cantaloube S, Romeo K, Le Baccon P, Almouzni G, Quivy J-P. 2012. Characterization of chromatin domains by 3D fluorescence microscopy: An automated methodology for quantitative analysis and nuclei screening. *Bioessays* **34**: 509–517.

Chan YW, Fava LL, Uldschmid A, Schmitz MHA, Gerlich DW, Nigg EA, Santamaria A. 2009. Mitotic control of kinetochore-associated dynein and spindle orientation by human Spindly. *J Cell Biol* **185**: 859-874.

Poullet P, Carpentier S, Barillot E. 2007. myProMS, a web server for management and validation of mass spectrometry-based proteomic data. *Proteomics* **7**: 2553-2556.
