# Supplementary figures and images for: The CENP-T/-W complex is a binding partner of the histone chaperone FACT
Source: Genes Dev. 2016 Jun 1;30(11):1313–26. doi: 10.1101/gad.275073.115 (PMC4911930; doi:10.1101/gad.275073.115)

**a.**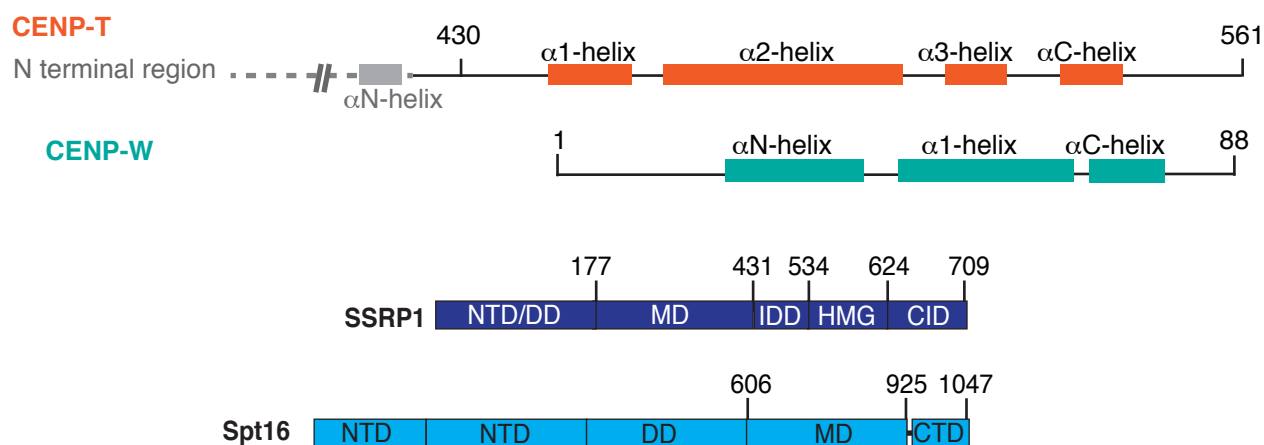**b.**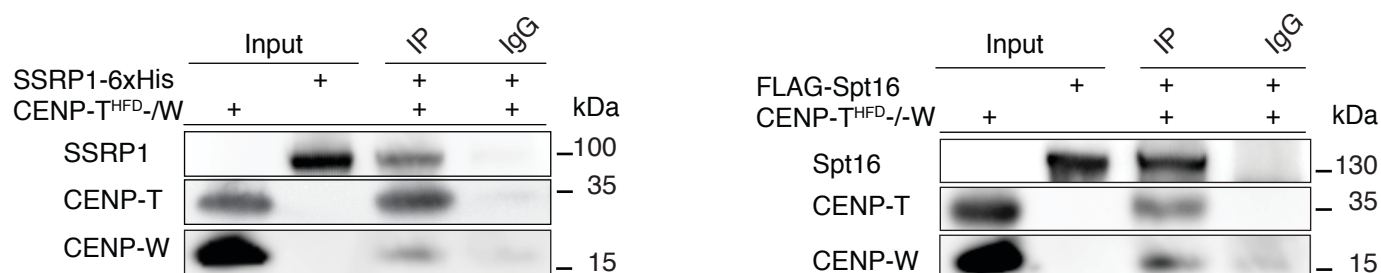**c.**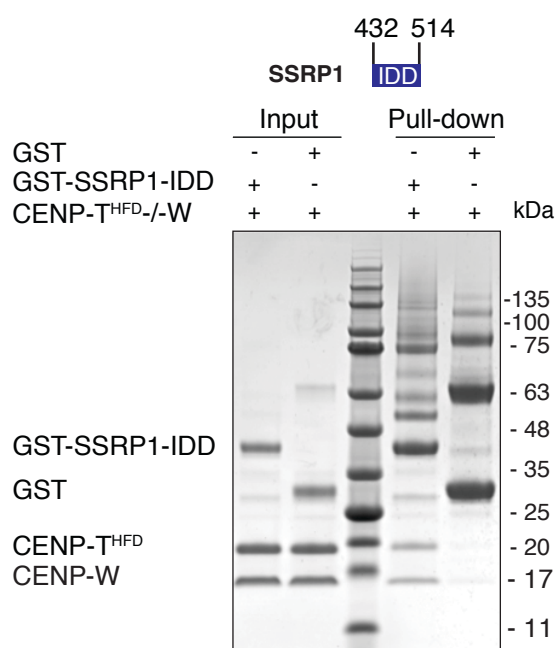**d.**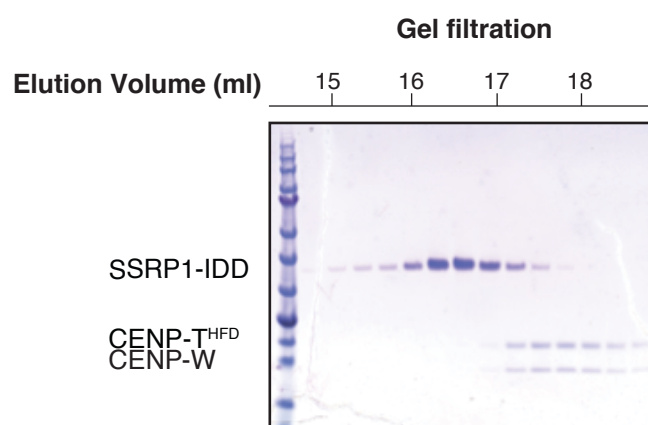

Supplement: Supplemental Material [file supp_gad.275073.115_Supplemental_FigS4.pdf]

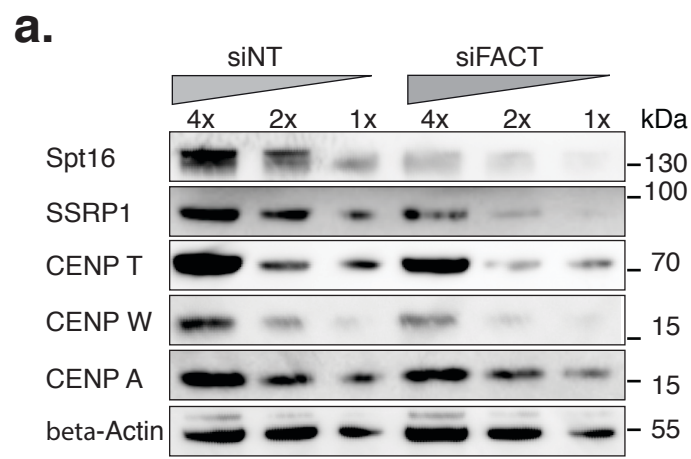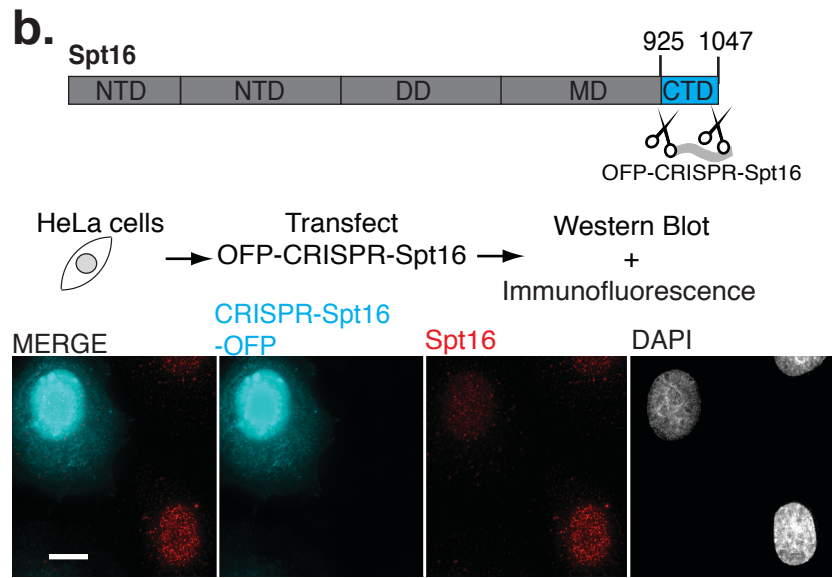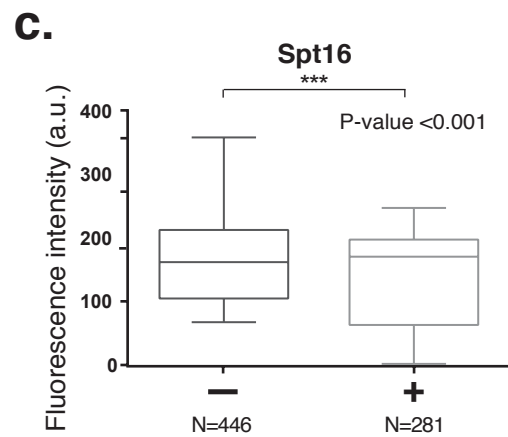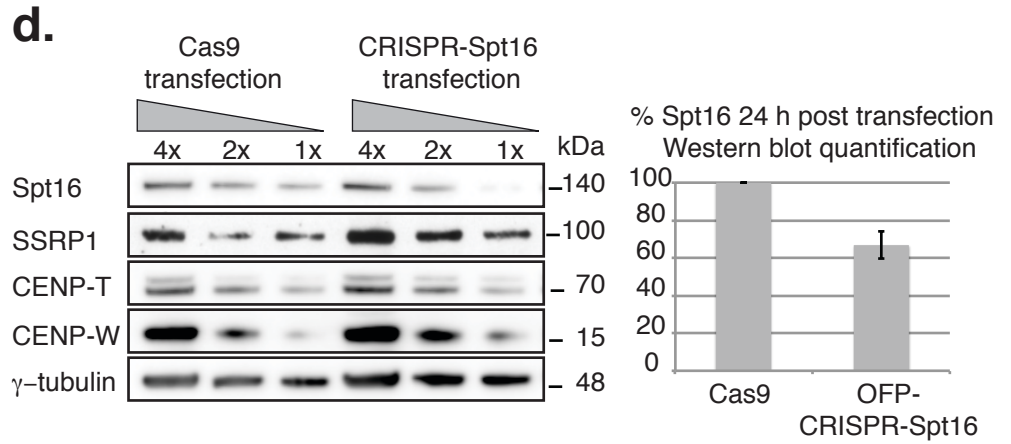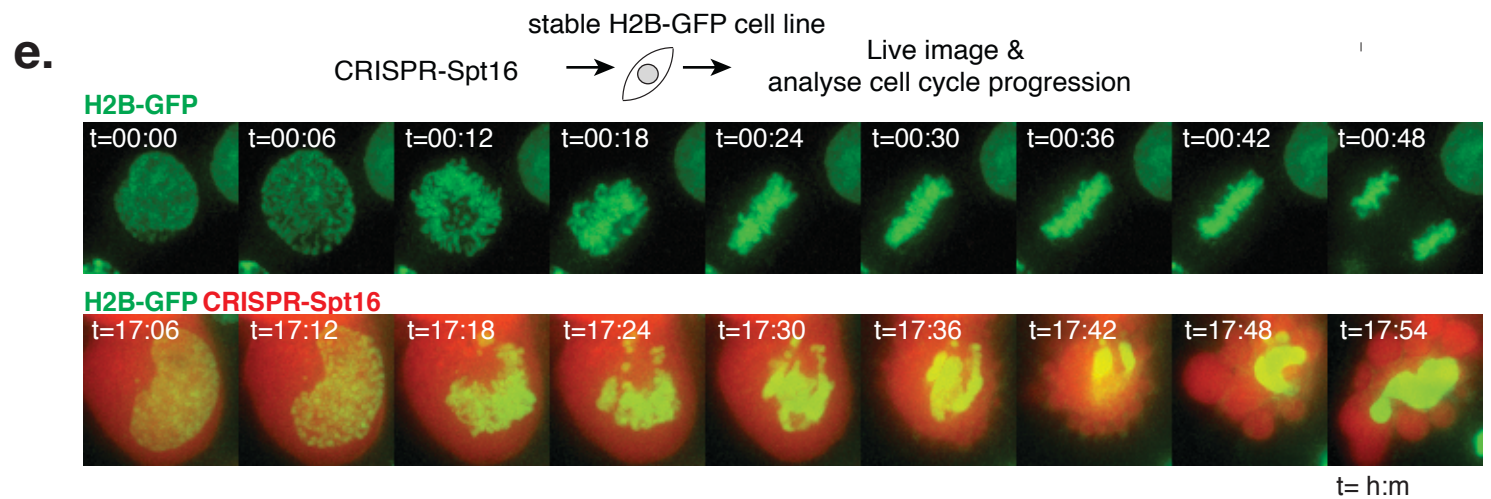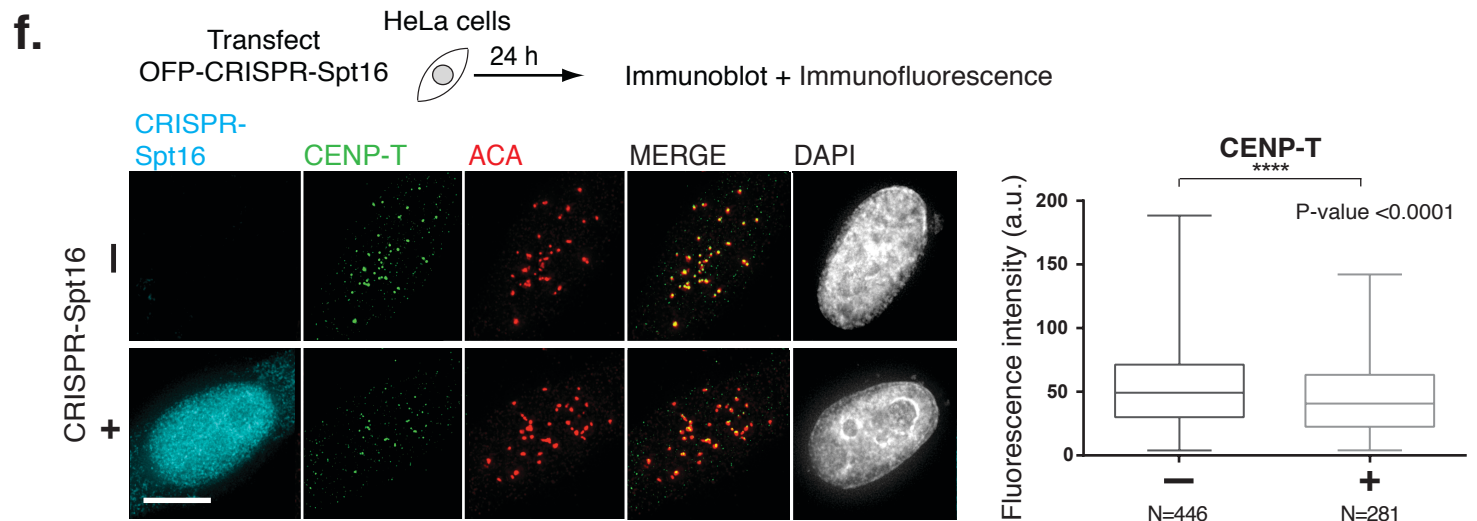

Supplement: Supplemental Material [file supp_gad.275073.115_Supplemental_FigS6.pdf]

**a.**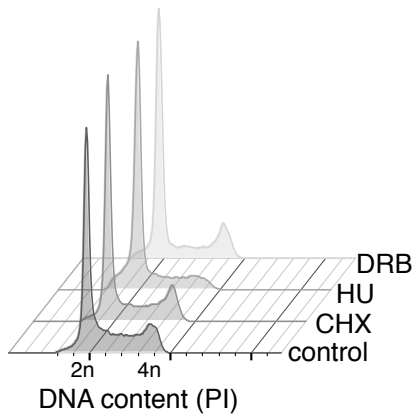**b.**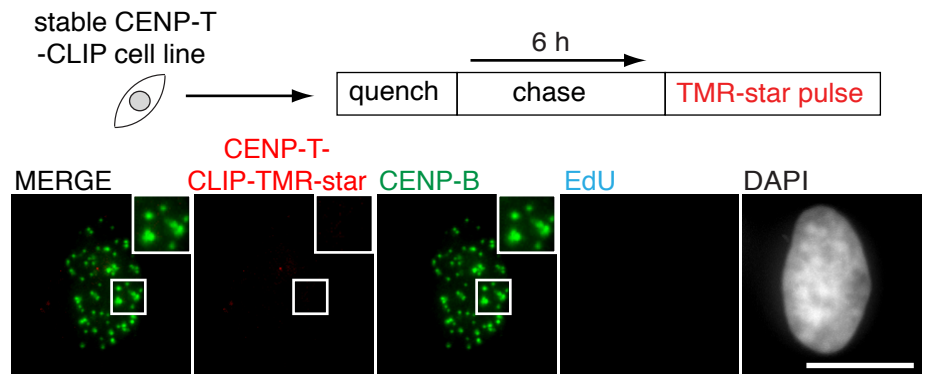**c.**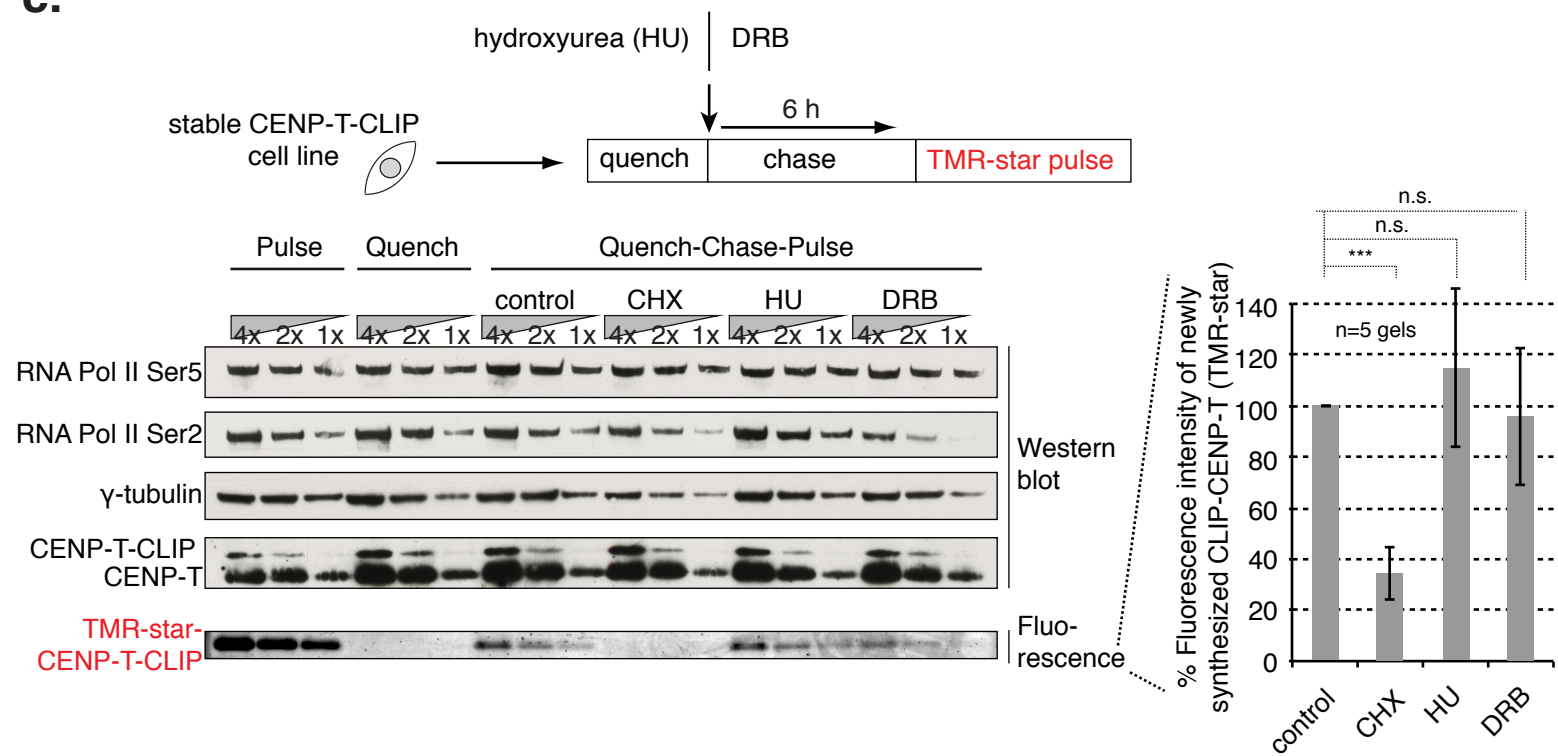**d.**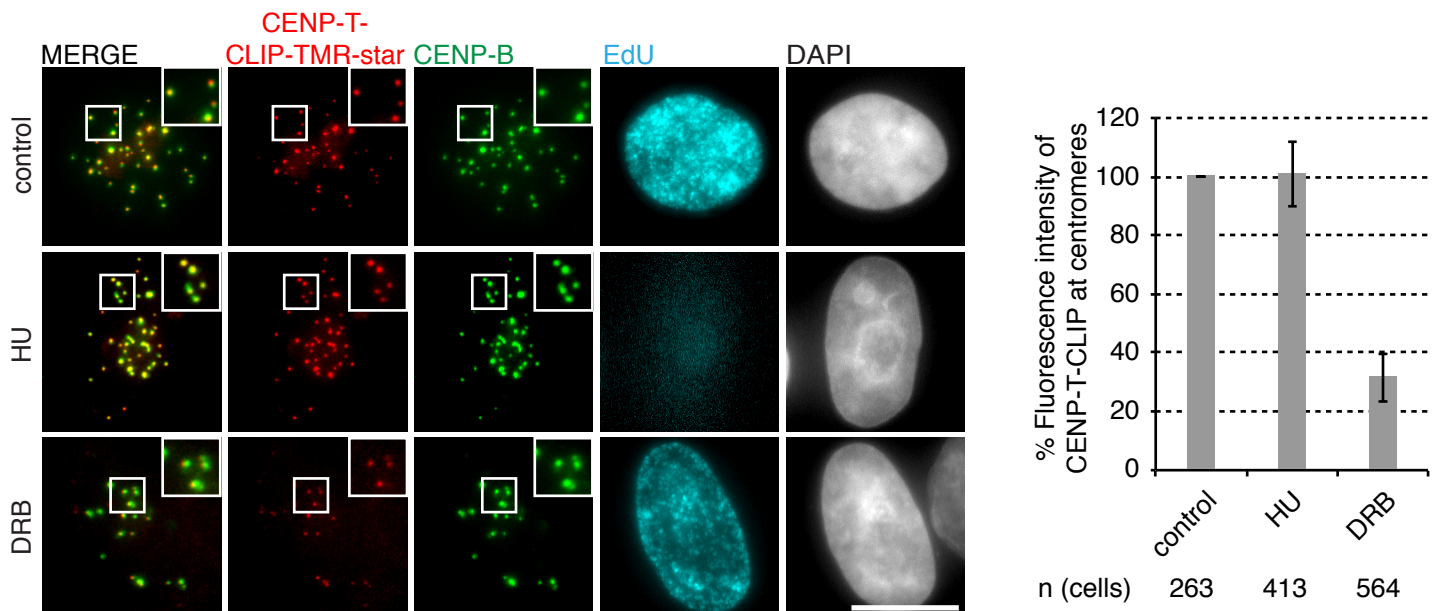

Supplement: Supplemental Material [file supp_gad.275073.115_Supplemental_FigS2.ps]

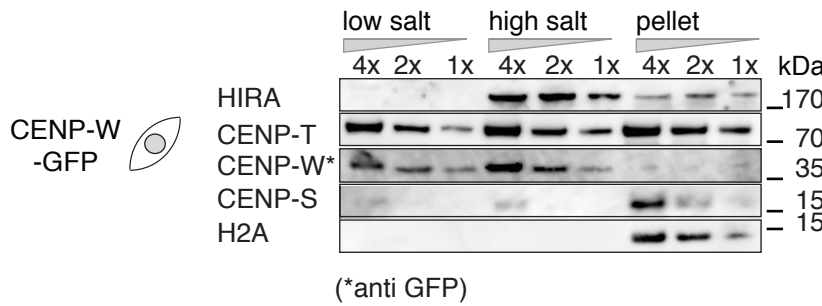

**b.**

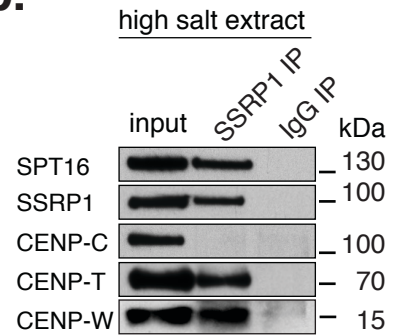

**C.**

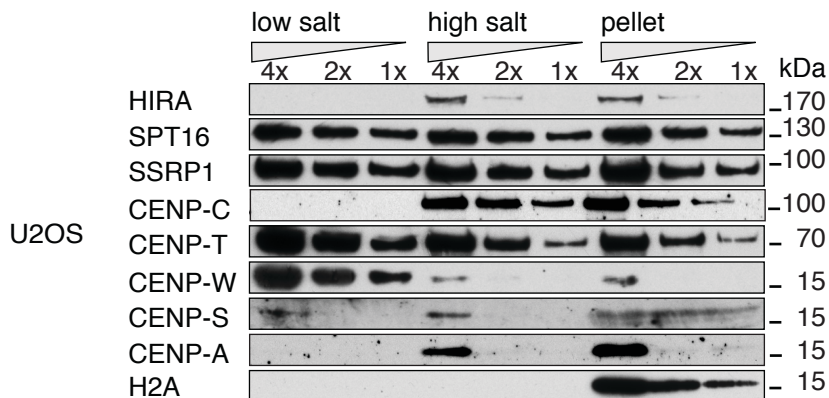

**d.**

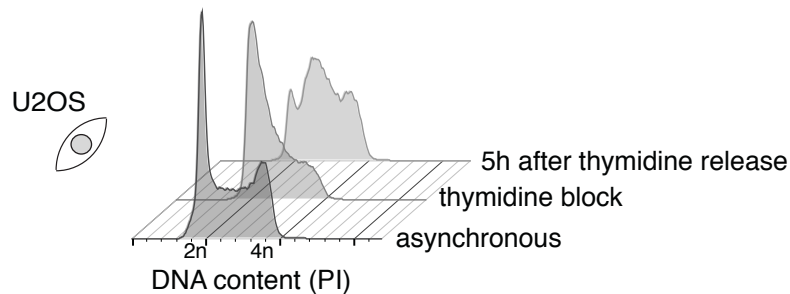

**e.**

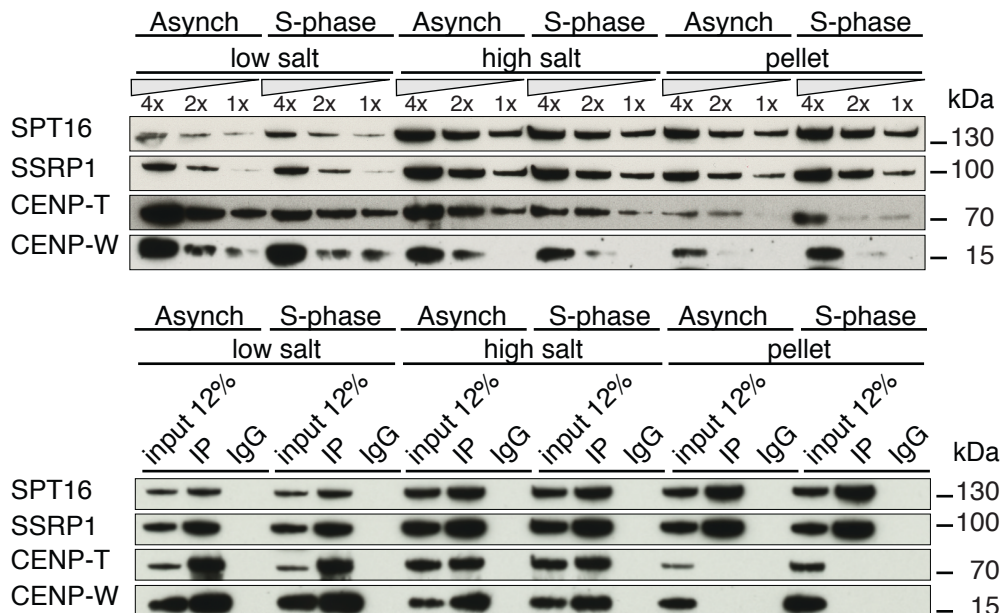

Supplement: Supplemental Material [file supp_gad.275073.115_Supplemental_FigS3.ps]

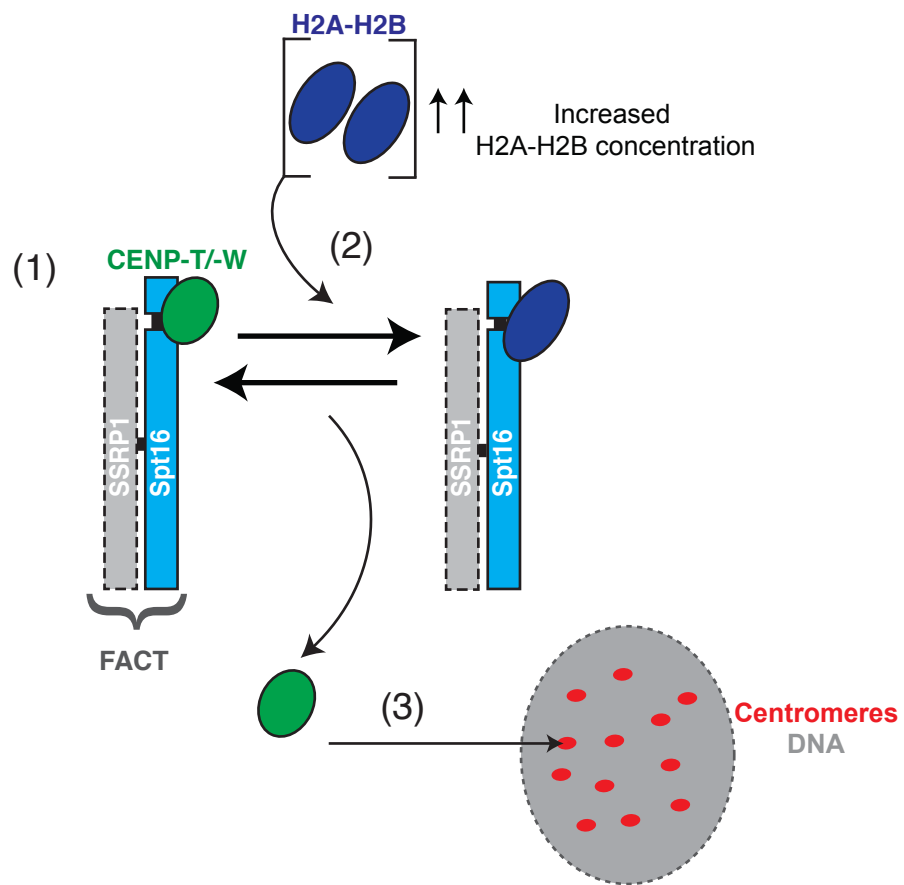

Supplement: Supplemental Material [file supp_gad.275073.115_Supplemental_FigS8.ps]

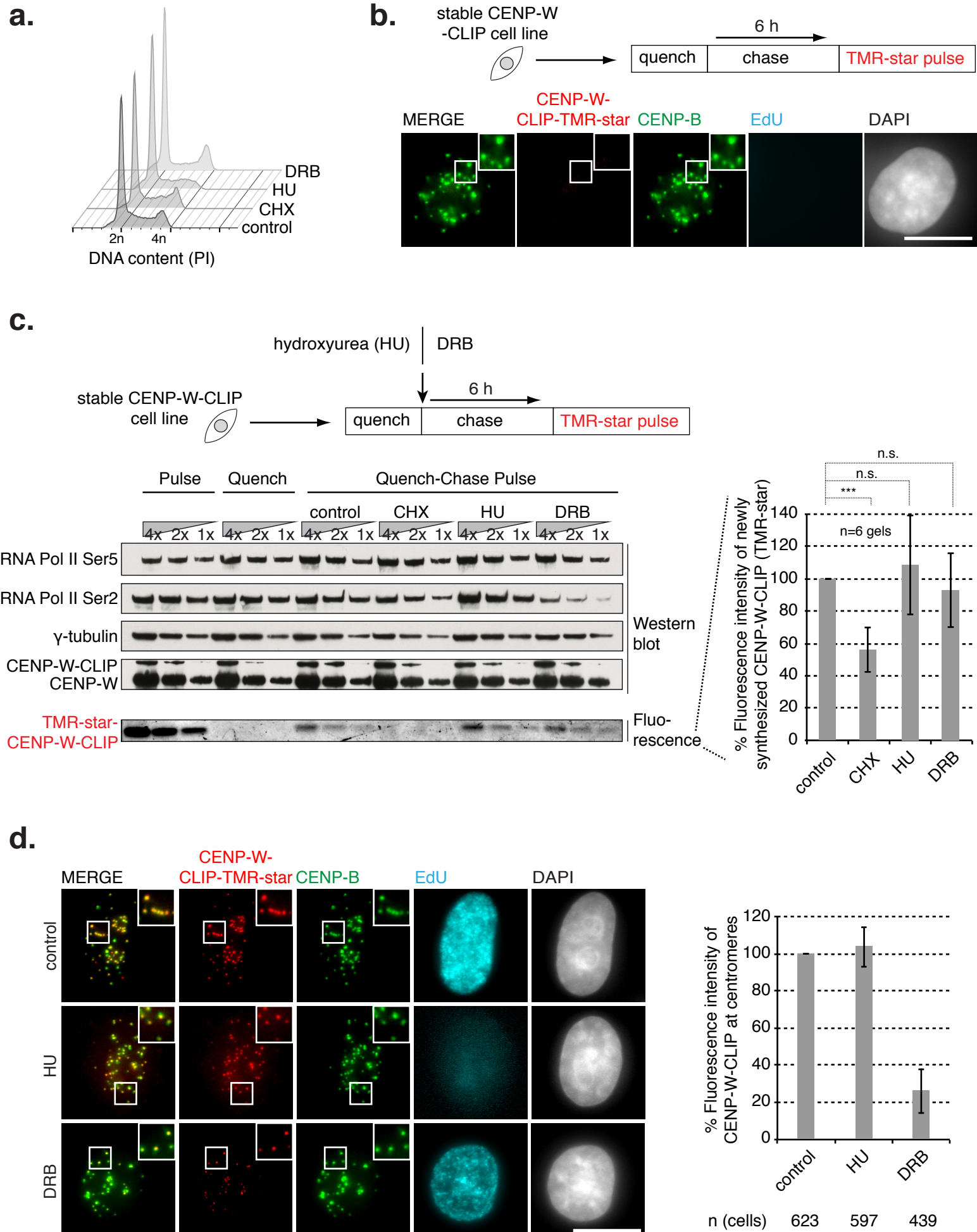

Supplement: Supplemental Material [file supp_gad.275073.115_Supplemental_FigS1.ps]

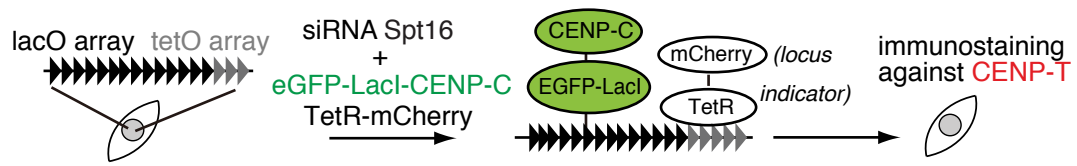

Count % of eGFP-LacI-CENP-C-LacO  
positive cells which recruit CENP-T

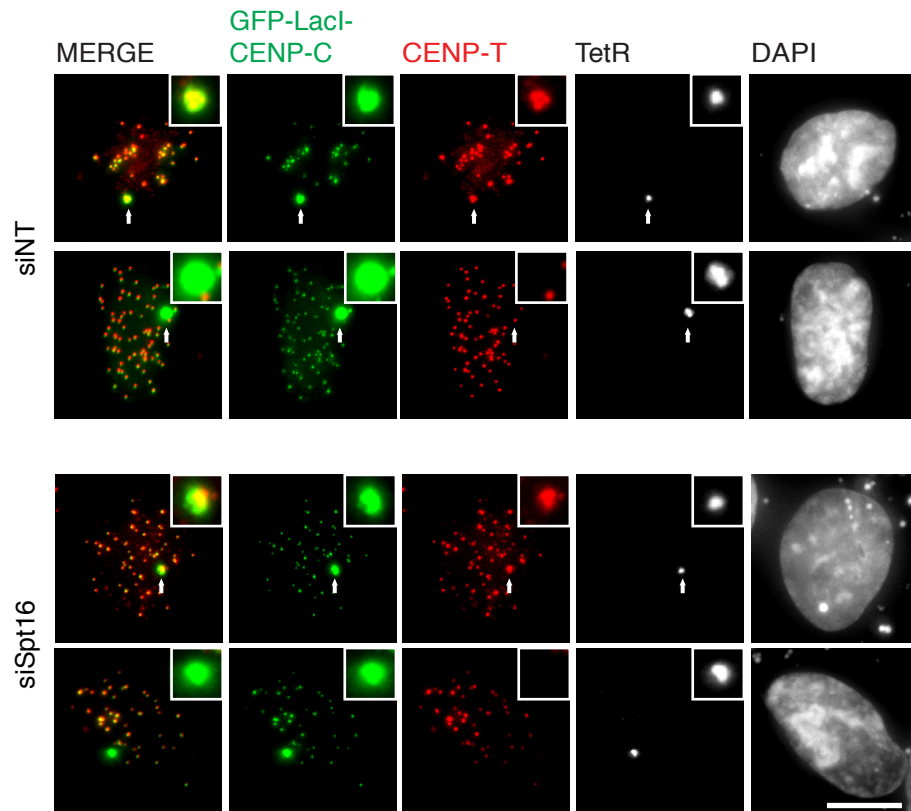

Supplement: Supplemental Material [file supp_gad.275073.115_Supplemental_FigS7.ps]
