# Supplementary material for: The CENP-T/-W complex is a binding partner of the histone chaperone FACT
Source: Genes Dev. 2016 Jun 1;30(11):1313–26. doi: 10.1101/gad.275073.115 (PMC4911930; doi:10.1101/gad.275073.115)

Prendergast_FigS5

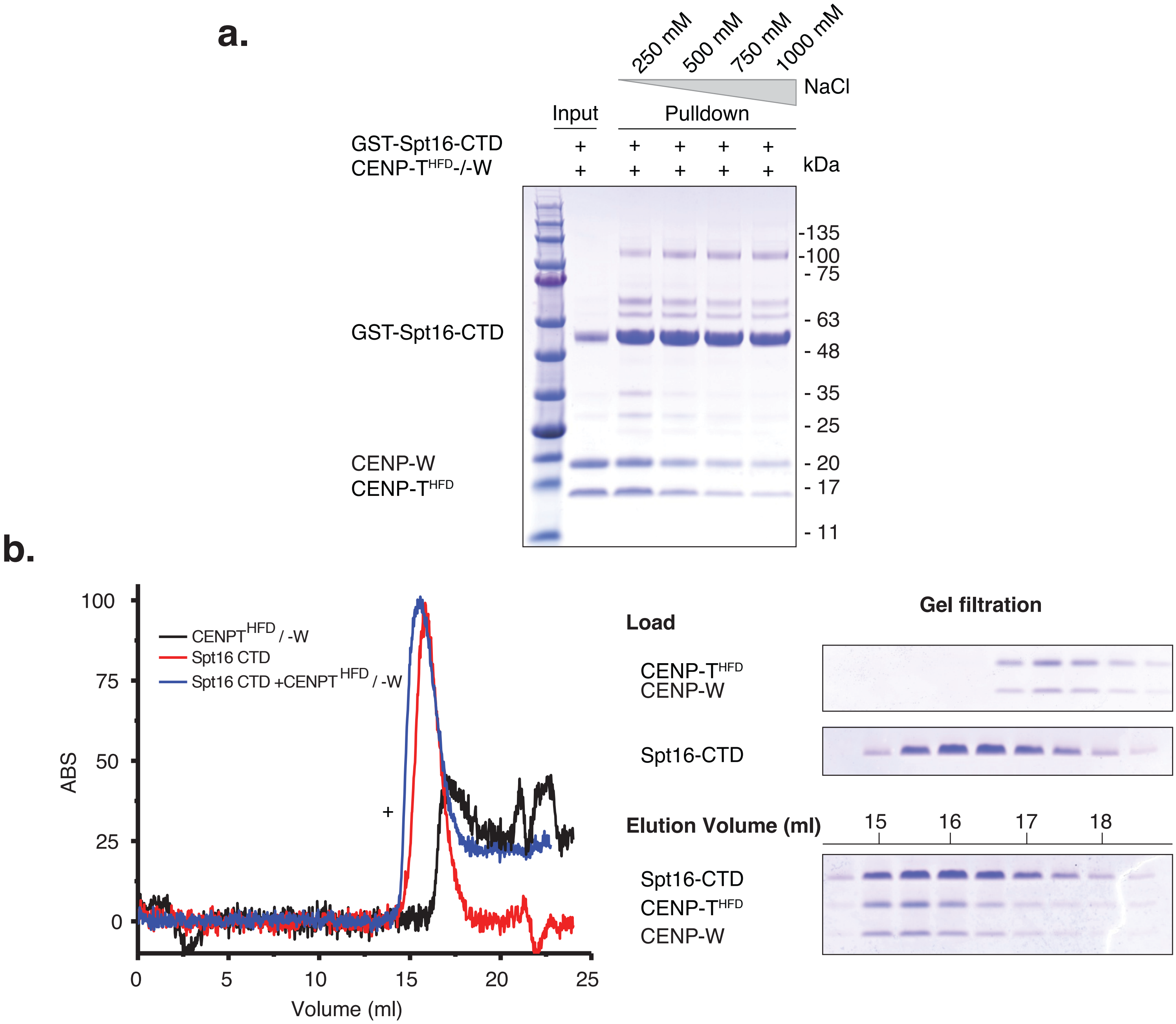

Supplement: Supplemental Material [file supp_gad.275073.115_Supplemental_FigS5.pdf]
