# Supplementary material for: The CENP-T/-W complex is a binding partner of the histone chaperone FACT
Source: Genes Dev. 2016 Jun 1;30(11):1313–26. doi: 10.1101/gad.275073.115 (PMC4911930; doi:10.1101/gad.275073.115)
Supplement: Supplemental Material [file supp_gad.275073.115_Supplementary_Figure_Legends.docx]

**Supplementary Figure Legends**

**Supplemental Table 1**

List of putative CENP-W-GFP interactors uncovered by mass spectrometry. GFP pull-downs were carried out, in biological triplicate, on control (GFP control, transient transfection of HeLa cells) and sample (CENP-W-GFP) populations using both low salt and high salt extracts. A cut off of >3 peptides per protein was used for inclusion in the protein list. Hits are ordered by peptide count. We detect some Spt16 peptides in GFP controls analyzed by MS.

**Figure S1: Related to Figure 1**

a. FACS analyses were used to assay the effect of DRB or HU treatment on cell cycle progression in CENP-W-CLIP cells.

b. An illustrative example of an EdU negative cell taken from a CENP-W-CLIP Quench-Chase-Pulse experiment.

c. Experimental scheme outlines approach. A Quench-Chase-Pulse assay was used to visualize ‘new’ TMRStar labelled CENP-W-CLIP centromere assembly in the presence of HU, DRB or cycloheximide. Newly synthesized protein was visualized by fluorescence scanning of an SDS-PAGE gel. 6 independent gels were quantified to calculate the level of total newly synthesized CENP-W-CLIP protein levels.

d. A Quench-chase-Pulse assay was used to visualize ‘new’ CENP-W-CLIP centromere assembly in the presence of HU or DRB. Centromeres were co-stained with anti-CENP-B antibodies. The fluorescence intensities of CENP-W-CLIP signal at centromeres were quantified in 3D (see materials and methods). Images were taken at 60x magnification.

**Figure S2: Related to Figure 1**

a. FACS analyses were used to assay the effect of DRB or HU treatment on cell cycle progression in CENP-W-CLIP cells.

b. An illustrative example of an EdU negative cell taken from a CENP-T-CLIP Quench-chase-Pulse experiment.

c. Experimental scheme outlines approach. A Quench-Chase-Pulse assay was used to visualize ‘new’ TMRStar labelled CENP-T-CLIP centromere assembly in the presence of HU, DRB or cycloheximide. Newly synthesized protein was visualized by fluorescence scanning of an SDS-PAGE gel. 5 independent gels were quantified to calculate the level of newly synthesized CENP-T-CLIP protein levels.

d. A Quench-chase-Pulse assay was used to visualize ‘new’ CENP-W-CLIP centromere assembly in the presence of HU or DRB. Centromeres were co-stained with anti-CENP-B antibodies. The fluorescence intensities of CENP-T-CLIP signal at centromeres were quantified in 3D (see materials and methods). Images were taken at 60x magnification.

**Figure S3: Related to Figure 2**

a. Fractionated protein extracts were prepared from cells expressing CENP-W-GFP following the scheme outlined in the Materials and Methods.

b. Western blot of SSRP1 co-IPs from CENP-W-GFP expressing cells confirms complex formation between SSRP1, Spt16 and CENP-T/-W.

c. Fractionated protein extracts were prepared from U2OS cells following the scheme outlined in the Materials and Methods.

d. FACS analyses of U2OS cells to assess synchrony following Thymidine arrest.

e. Western blot of SSRP1 co-IPs from low-salt, high-salt and pellet extracts prepared from asynchronous and S-phase synchronized U2OS cells. Spt16, CENP-T and CENP-W co-IP with SSRP1 from low-salt and high-salt extracts, but not in the pellet fraction.

**Figure S4: Related to Figure 3**

a. Schematic illustrates the region of the CENP-T histone fold region (CENP-T^HFD^ (aa430-561)) that was co-expressed with full length CENP-W (aa1-88) and purified (see Materials and Methods).

b. CENP-T^HFD^/-W was used as bait in an *in vitro* binding experiment. Either recombinant Spt16-FLAG or SSRP1-His were incubated with CENP-T^HFD^/-W in IP buffer + 0.1% BSA (see Materials and Methods). Resulting complexes were purified by IP with anti-CENP-T antibodies or rabbit IgG control antibodies. Western blots on IPs show Spt16, SSRP1, CENP-T and CENP-W.

c. GST-SSRP1-IDD was used as bait for an *in vitro* GST pull-down assay with CENP-T^HFD^/-W. GST protein was used as bait in control pull-downs. Coomassie stained gel shows inputs and GST pull-downs.

d. GST-SSRP1-IDD and CENP-T^HFD^/-W do not form a stable complex when combined and subjected to gel filtration. The coomassie gel shows the elutions from a Superdex 200 10/300 GL analytical gel filtration column

**Figure S5: Related to Figure 3**

a. The interaction between Spt16-CTD and CENP-T^HFD^-CENP-W is stable in up to 1 M NaCl. GST-Spt16-CTD was incubated with excess amount of CENP-T^HFD^-CENP-W in binding buffer (20 mM Tris-HCl pH 7.5, 250 mM NaCl and 0.1 mM DTT) for 1 h, and washed 5 x with washing buffer containing different salt concentrations (20 mM Tris-HCl pH 7.5, 0.1 mM DTT, 2% Triton x-100, and 250 mM NaCl for sample 1, 500 mM NaCl for sample 2, 750 mM NaCl for sample 3, 1000 mM NaCl for sample 4). The resulting samples were analyzed with SDS-PAGE.

b. Spt16-CTD and CENP-T^HFD^-CENP-W form a stable complex when combined, and co-elute from an analytical gel filtration column Superdex 200 10/300 GL. Running buffer: 20 mM Tris-HCl pH 7.5, 250 mM NaCl and 0.1 mM DTT. Purified Spt16-CTD and CENP-T^HFD^-CENP-W were mixed at 1:1 ratio and incubated 1 h before loading onto a gel filtration column. They co-migrated on the column. Purified CENP-T^HFD^-CENP-W and Spt16-CTD alone were used as control.

**Figure S6: Related to Figure 5**

a. Cells were depleted of FACT by siRNA against SSRP1 and Spt16

for 48 h. The levels of proteins of interest were assayed by Western blot.

b. Experimental scheme outlines the CRISPR approach. CRISPR-Spt16 plasmids carrying an OFP fluorescent reporter were transfected into cells stably expressing CENP-W-GFP or WT HeLa. Spt16 nuclear signal was visualized by IF using anti-Spt16 antibodies. OFP has been false-colored to Cyan using ImageJ for clarity.

c. Spt16 fluorescence intensity was measured in individual CRISPR-Spt16 transfected cells and found to be significantly reduced when compared to untransfected cells (P value >0.001 Kolmogorov-Smirnov test).

d. Western blot analysis of CRISPR-Spt16 transfected cells shows relative levels of Spt16, CENP-T and CENP-W.

e. Scheme describes experimental approach. HeLa cells stably expressing H2B-GFP were transfected with CRISPR-Spt16 plasmids. Starting from 6 h following transfection, cells were imaged every 6 minutes. Images are stills from live cell movie (Supplemental Movie S1).

f. Centromere intensities of CENP-T were quantified in all cells expressing CRISPR-Spt16 and cells not cells expressing CRISPR-Spt16. Anti-centromere antibody (ACA) was used to identify centromeres. Quantification was performed in 3D on deconvolved Z stacks using an automated ImageJ macro (see Experimental procedures). Experiments were repeated 4 times. Graphs shown are Min to Max plots of all centromere intensities collected. A minimum of 30 cells were quantified per experiment, n = number of cells quantified. P-value for significance is calculated using a 2-tailed student T-test. Maximum intensity projections were used to prepare images. Scale bar =10 μM.

**Supplemental Movie S1**

CRISPR-Spt16 plasmids carrying orange fluorescent protein (OFP) reporter were transfected into HeLa cells stably expressing H2B-GFP. Cells were imaged every 6 minutes starting from 6h after transfection. Movie S1 shows a transfected cell undergo apoptosis. The movie time stamp indicates time of imaging. At T=0, cells were 6h post transfection.

**Figure S7: Spt16 is sufficient for *de novo* CENP-T accumulation**

Schematic outlines LacO-LacI tethering approach. A cell line stably expressing a LacO-TetR array was transfected with siRNA (siNT or siSpt16) for 24 h. Following 24 h, siRNA treated cells were transiently transfected with eGFP-LacI-CENP-C and TetO-mCherry. Cells were then fixed after a further 24 h and IF for CENP-T was performed. TetR-mCherry allows visualization of the LacO tethered eGFP-LacI-CENP-C. We counted the number of cells in each experiment that recruited CENP-T (Figure 6a). The immunofluorescence images show examples of cells with or without CENP-T recruitment in both siSpt16 treated samples and siNT treated samples.

**Figure S8: Graphical Scheme**

(1) The Spt16 subunit of the FACT complex binds CENP-T/-W through the Spt16-CTD. FACT is recruited to chromatin (possibly in the context of transcription and concomitant with replication). The FACT-CENP-T/-W interaction excludes H2A-H2B and thus may occur when soluble H2A-H2B is sequestered by a dedicated chaperone or actively used in transcription related events.

(2) H2A-H2B competes with CENP-T/-W for Spt16 binding. The release of stably bound CENP-T/-W from Spt16 would then occur through competition or exchange with H2A-H2B when H2A-H2B concentration is increased. H2A-H2B concentration may be increased in proximity to chromatin.

(3) CENP-T/-W may be incorporated at centromeres once released from FACT. If CENP-T/-W is released outside centric regions, no stable chromatin incorporation of CENP-T/-W should occur because of the unfavorable competition and absence of retention mechanisms. If CENP-T/-W is released in proximity to centric regions CENP-T/-W may readily bind to centromeric CENP-T/-W already present, or to CENP-C. This retention could ultimately result in CENP-T/-W accumulation.
